# Tumor growth suppression in adoptive T cell therapy via IFN-γ targeting of tumor vascular endothelial cells

**DOI:** 10.7150/thno.101107

**Published:** 2024-10-21

**Authors:** Qiaoya Lin, Colleen P. Olkowski, Peter L. Choyke, Noriko Sato

**Affiliations:** Molecular Imaging Branch, Center for Cancer Research, National Cancer Institute, National Institutes of Health, Bethesda, Maryland, USA.

**Keywords:** adoptive T cell therapy, Interferon-gamma, tumor vessel endothelial cells, endothelial cell regression, tumor hypoxia

## Abstract

**Rationale:** In adoptive T cell therapy (ACT), the direct cytotoxic effects of CD8 T cells on tumor cells, including the release of interferon-gamma (IFN-γ), are considered the primary mechanism for tumor eradication. Cancer antigen escape diminishes the T cell responses, thereby limiting the therapeutic success. The impacts of IFN-γ targeting non-tumor cells in ACT, on the other hand, remains under-investigated. We hypothesized that IFN-γ action on non-tumor cells, particularly tumor vascular endothelial cells within the physiological tumor microenvironment, could influence therapeutic efficacy.

**Methods:** ACT was performed against ovalbumin (OVA)- or OVA-peptide SIINFEKL-expressing syngeneic mouse tumors, MCA-205-OVA-GFP fibrosarcoma or MOC2-SIINFEKL oral squamous cell carcinoma, using *ex vivo*-activated OT-1 CD8 T cells expressing the T cell receptor against OVA. Efficacy was examined in wild-type mice, mice deficient for IFN-γ receptor 1 (IFN-γR1KO), and bone marrow chimeras lacking IFN-γR1 expression in endothelial cells. To exclude direct IFN-γ action against tumor cells, IFN-γR1KO-MCA-205-OVA-GFP tumors were used. IFN-γ production, STAT1 induction in its targets, and subsequent changes, especially in vasculatures in the tumor, were examined.

**Results:** ACT suppressed the growth of MCA-205-OVA-GFP and MOC2-SIINFEKL tumors in wild-type mice but failed in IFNγR1KO mice. Furthermore, in the bone marrow chimeras lacking endothelial cell IFN-γR1, ACT efficacy was lost, thus implicating a vital role of IFN-γ action on the endothelium. IFN-γR1KO-MCA-205-OVA-GFP tumor growth was successfully suppressed by ACT in wild-type mice, suggesting that IFN-γ targeting of tumor cells may not be essential for ACT efficacy. OT-1 CD8 T cells interacted with endothelial cells or localized in proximity to the vessels on Day 1.5 after transfer, as observed by intravital microscopy. The OT-1 T cells found in tumors were limited in number but produced high levels of IFN-γ on Day 1.5, while their number peaked on Day 5.5 with negligible IFN-γ production. Together with IFN-γ production by endogenous lymphocytes, IFN-γ levels in the whole tumor peaked on Day 1.5, inducing IFN-γ/STAT1 signaling in endothelial cells. Early targeting of tumor vascular endothelial cells by IFN-γ led to endothelial regression, reduced perfusion, and tumor hypoxia/necrosis (Day 4.5-7).

**Conclusions:** These findings highlight the critical role of T cell-derived IFN-γ action on endothelial cells early in ACT, emphasizing its dynamic influence on the tumor microenvironment, and offering insights into addressing antigen escape.

## Introduction

Adoptive T cell therapy (ACT) has become a major immunotherapeutic strategy since the emergence of genetically engineered cancer-antigen specific T cells, such as chimeric antigen receptor-T (CAR-T) cells and T cell receptor (TCR) transduced T (TCR-T) cells 1, 2. T cells used in these therapies are harvested, genetically modified, and expanded *ex vivo* before they are infused back into the patient. Tumor-infiltrating lymphocytes (TILs) have also been used as a source of tumor-specific T cells that do not require genetic modifications. Although ACT has demonstrated favorable outcomes in hematological malignancies, it has met with less success in solid tumors 1, 3. Possible explanations include limited tumor infiltration of T cells, antigen escape related to the highly heterogeneous landscape of tumor-associated antigens, exhaustion of T cells, and the intricacies of the tumor microenvironment including the presence of various immune suppressive cells 1, 3-5.

In ACT, CD8 T cells infiltrating the tumor microenvironment are considered directly cytotoxic while CD4 T cells have an ancillary role. It is well-established that cytotoxic CD8 T cell-released granzymes or perforin directly kill tumor cells following engagement of the major histocompatibility complex class I (MHC-I)/TCR in the context of T cell recognition of presented tumor antigen. CD8 T cells release a wide range of cytokines and chemokines upon activation 6, 7, among which, interferon-γ (IFN-γ) is considered one of the most important. IFN-γ is thought to exert either a direct cytotoxic effect or, at least, cytostatic effects on tumor cells 8, 9, contributing to tumor senescence 10 or ferroptosis 10, 11. IFN-γ receptor deficiency in glioblastoma and other solid tumors increases resistance to CAR-T cell-mediated killing 12. The direct cytotoxic effects by CD8 T cells, particularly those mediated by IFN-γ, on tumor cells constitute the primary mechanism for tumor eradication in ACT. In contrast, whether IFN-γ action on non-tumor cells affects the efficacy of ACT and the mechanism involved remain under-investigated. IFN-γ released by CAR-T cells activates myeloid cells and induces endogenous memory T cell responses 13. Additionally, IFN-γ-dependent anti-angiogenesis has been reported as a mechanism in tumor rejection by CD8 and CD4 effector T cells 14. Tumor vessel reduction has been observed on Day 5 after ACT in mouse tumor models 15, 16; however, the underlying mechanisms are unknown. Whether and how IFN-γ affects non-tumor cell components, especially vasculatures, in a physiological tumor microenvironment and resulting ACT outcome remain to be further elucidated. Furthermore, the effects of IFN-γ are dose and spatiotemporally dependent 17, 18. Studies indicate that low-dose IFN-γ can induce metastatic properties in tumors, while high-dose IFN-γ leads to tumor regression. Thus, the concentration of IFN-γ within the tumor microenvironment could determine its functional outcome.

This study aims to understand the effects of IFN-γ on the tumor microenvironment during ACT, with a focus on tumor vascular endothelial cells. Using mouse ACT models against syngeneic cancers, we demonstrate the critical role of IFN-γ receptor 1 (IFN-γR1) on endothelial cells in the efficacy of ACT. We reveal that *ex vivo* activated T cells, although limited in cell number, produce high levels of IFN-γ early after ACT, triggering regression of endothelial cells, ultimately leading to hypoxia/necrosis. This finding could be an important clue for designing novel strategies to avoid antigen escape during ACT.

## Methods

### Mice

C57BL/6J, B6.SJL-*Ptprc^a^Pepc^b^*/BoyJ (Ly5.1), B6.PL-*Thy1^a^*/CyJ (Thy1.1), B6.129S7-*Ifngr1^tm1Agt^*/J (IFN-γ receptor-deficient: IFNγR1KO), B6.Cg-Tg(Tek-cre)12Flv/J (Tie2^Cre^), C57BL/6N-*Ifngr1^tm1.1Rds^*/J (IFNγR1^flox/flox^), B6.129(Cg)-*Gt(ROSA)26Sor^tm4(ACTBtdTomato-EGFP)Luo^*/J (mTmG), B6.129S4-*Ifng^tm3.1Lky^*/J (GREAT), and mice transgenic for ovalbumin (OVA)-specific TCR C57BL/6-Tg(TcraTcrb)1100Mjb/J were obtained from Jackson Laboratory. The C57BL/6-Tg(TcraTcrb)1100Mjb/J mice were crossed to Thy1.1 mice or mTmG mice to generate OT-1 mice that express Thy1.1, or mTmG-OT-1 mice (express Thy1.2), respectively. GREAT-OT-1-Thy1.1 mice were generated by crossing GREAT mice with OT-1 mice. Tie2^Cre^ mice and IFNγR1^flox/flox^ mice were crossed to generate Tie2^Cre^IFNγR1^flox/flox^ mice. Male and female mice aged 10-24 weeks were used in the experiments. All mouse experiments were conducted in accordance with animal protocol (MIP-008) approved by the National Cancer Institute Animal Care and Use Committee.

### Bone marrow chimera mice

Donor bone marrow cells were isolated from femurs and tibias of Ly5.1 mice. The cells were intravenously injected into lethally irradiated Tie2^Cre^IFNγR1^flox/flox^ mice or littermate IFNγR1^flox/flox^ mice expressing Ly5.2 within 6 h of irradiation (9.5 Gy using a cesium-137 irradiator GammaCell 40, Atomic Energy of Canada). After 8 weeks, mice with more than 95% chimerism in peripheral blood as determined by flow cytometry analysis, were selected for further experiments.

### Tumor cell lines and subcutaneous tumor models

The syngeneic MCA-205-OVA-GFP (murine fibrosarcoma) cells expressing ovalbumin was a gift from Zhiya Yu, National Institutes of Health (originally from Andrea Schietinger), and IFN-γR1KO-MCA-205-OVA-GFP cells were generated via using CRISPR techniques to knock out IFN-γR1 (Applied Biological Materials Inc.). MOC2-SIINFEKL (murine oral squamous cell carcinoma) cells expressing ovalbumin peptide SIINFEK was a gift from John S. Davies, National Institutes of Health. The cells were cultured in RPMI1640 medium (Thermo Fisher Scientific) supplemented with 10% fetal bovine serum (Gemini), 100 IU/mL penicillin, 100 μg/mL streptomycin (Thermo Fisher Scientific), and 0.05 mM 2-mercaptoethanol (Sigma). Subcutaneous tumors were established by inoculating 1.0 × 10^6^ MCA-205-OVA-GFP or IFN-γR1KO-MCA-205-OVA-GFP cells, or 2.0 × 10^6^ MOC2-SIINFEKL cells, suspended in 100 μL phosphate-buffered saline (PBS, Corning) into the right flank of C57BL/6J or mTmG mice. Tumor size was measured using a caliper and the tumor volume (V) was calculated using the formula; V = (major axis) × (minor axis)^2^ × ½. The tumors were monitored until the major axis reached 20 mm. Mice harboring tumors with approximate volumes of 100~200 mm^3^ were randomly assigned to experimental groups.

### Adoptive T cell therapy

Splenocytes derived from OT1-Thy1.1, mTmG-OT-1, or GREAT-OT1-Thy1.1 mice were activated by culturing the cells with 1 μg/mL OVA peptide (SIINFEKL, AnaSpec) and 0.3 nM human interleukin-2 (Peprotech) for 2.5 - 3 days. Subsequently, the obtained CD8 T cells were washed with PBS and 3.0 × 10^6^ cells were administered intravenously to mice bearing subcutaneous tumors.

### Flow cytometry analyses

To analyze cells in the tumors, harvested tumors were minced into small pieces using scissors, digested with 50 μg/mL Liberase TM (Roche) at 37 °C for 30 min in complete medium, and passed through a 70 μm filter. These single-cell suspensions were centrifuged, and the cells were then resuspended in PBS. After centrifugation, the cells were incubated with an anti-CD16/32 antibody to block Fc binding and, subsequently, with fluorophore-conjugated antibodies in PBS with 1% fetal bovine serum and 1 mM ethylenediaminetetraacetic acid (Phoenix BioTechnologies,) for 30 min at 4 °C. Intracellular IFN-γ staining of surface-stained cells was performed using a Foxp3/Transcription Factor Staining Buffer Kit (Thermo Fisher Scientific) following the manufacturer's instructions. The stained samples were applied to a CytoFLEX flow cytometer (Beckman Coulter) and obtained data were analyzed using the FlowJo software (v10.8, BD Life Sciences). The delta mean fluorescence intensity (ΔMFI) was calculated as follows: ΔMFI = ∑geometric mean of sample -∑geometric mean of isotype control.

To determine the bone marrow chimerism, blood samples collected from the mice 8 weeks after the bone marrow transfer were layered on the lymphocyte separation medium (Promo Cell GmbH) and centrifuged to collect the peripheral mononuclear cells in the interphase. Obtained cells were stained with anti-Ly5.1 and Ly5.2 antibodies for 10 min at room temperature and applied to the flow cytometer. See Table S1 for detailed information of the antibodies used.

### Intravital imaging

Short-term *in situ* intravital imaging experiments were conducted on various days following the GREAT-OT-1-Thy1.1 CD8 T cell therapy of MCA-205-OVA-GFP tumor in mTmG mice using the ZEISS LSM 880 laser scanning confocal microscope (Carl Zeiss). In brief, the epidermis was incised, and a cover slip was directly placed on the exposed tumor tissue for imaging of a maximum of 6 h (see Supplementary **materials and methods** for details). To detect infused GREAT-OT-1-Thy1.1 CD8 T cells in Thy1.2 expressing-mTmG recipient mice, allophycocyanin (APC)-conjugated anti-Thy1.1 antibody (6 µg) in 200 µL PBS was intravenously injected approximately 1 h prior to imaging. Similarly, mTmG-OT-1 CD8 T cells were used to treat MCA-205-OVA-GFP tumor in wild-type (WT) mice in conjunction with Alexa Fluor 647-conjugated anti-CD31 antibody (8 µg) injection to visualize tumor vessels which has perfusion. Intravital microscopy images were processed via Image J software (National Institutes of Health), 3D movies were generated using ZEN software (Carl Zeiss), employing the maximum intensity projection method.

### Multiplex chemokine/cytokine assay

MCA-205-OVA-GFP tumors were collected at specified time points ranging 1.5 days to 6.5 days following the indicated treatments, minced, and lysed in M-PER Mammalian Protein Extraction (Thermo Fisher Scientific, 50 µL per 1 mg of tissue) containing a protease inhibitor cocktail (Crystalgen) on ice. The lysed samples were centrifuged at 13,523 x* g* for 10 min at 4 °C, and the supernatants were collected and preserved at -80 °C until multiplex cytokine/chemokine assays were conducted by Eve Technologies (Calgary, Canada).

### Multiplex immunofluorescence imaging, hematoxylin and eosin staining, and image quantitation

MCA-205-OVA-GFP tumors collected 1.5-7 days following the T cell therapy or without therapy were fixed in 4% paraformaldehyde for 24-48 h and then transferred to 70% ethanol. Subsequently, 4 μm-thick formalin-fixed paraffin-embedded (FFPE) sections were prepared by American Histolabs, Inc. (Gaithersburg, MD). After deparaffinization, heat-induced antigen retrieval was performed and the multiplex immunofluorescence staining of FFPE sections was conducted using the Opal Automation IHC Kit (Akoya Bioscience) and the Bond RXm autostainer (Leica Biosystems). Hematoxylin and eosin (H&E) staining was performed by Histoserv, Inc. (Germantown, MD). Image acquisition was performed using the Mantra Quantitative Pathology Workstation with the Inform image analysis software (Perkin Elmer). Image J software (National Institutes of Health) was used for further image analysis and quantitation.

In some studies, 6 µg APC-anti-CD31 antibody in PBS was intravenously injected to label endothelial cells *in vivo*. At least 30 min later, mice were sacrificed, and the tumors were harvested for fixation in 4% PFA (Thermo Fisher Scientific) for 12-24 h, followed by transfer into a 30% sucrose solution (Fluka Analytical) for 24-48 h and then stored frozen. Additionally, 10 μm-thick sections were obtained using a Leica CM3050S cryostat. The frozen slides were mounted with a mounting medium containing 4',6-diamidino-2-phenylindole (DAPI, Thermo Fisher Scientific) and imaged using a ZEISS LSM 880 laser scanning confocal microscope (Carl Zeiss). Acquired images were analyzed using Image J software (National Institutes of Health).

### Transmission electron microscopy analyses

MCA-205-OVA-GFP tumors were collected at specified time points (1.5-7 days) following the T cell therapy and were cut into 1 mm^3^ pieces in an electron microscopy (EM) buffer consisting of 0.1M sodium cacodylate containing 4% formaldehyde and 2% glutaraldehyde. The tissues were fixed in the buffer for 2 h at room temperature and then stored at 4 °C until EM imaging at the Electron Microscopy Laboratory, Center for Cancer Research, National Cancer Institute, National Institutes of Health (Frederick, MD).

### *In vivo* and *ex vivo* blood perfusion imaging with IR800-conjugated albumin

Serum bovine albumin (1000 µg, Millipore Sigma) was incubated with 78.6 nM IRDye 800CW NHS ester (LI-COR Biosciences) in 300 μL of 0.1 M Na_2_HPO_4_ buffer (pH 8.5) for 60 min at room temperature. The resulting IR800-conjugated albumin was purified using a PD-10 Desalting Column (Cytiva) and PBS. For *in vivo* blood perfusion imaging of the tumor, 50 μg purified IR800-conjugated albumin in 150 μL PBS was intravenously administered to mice with their fur removed in the tumor area by shaving and Nair cream application (Church & Dwight Co). Whole-animal images capturing IR800 fluorescence were acquired 20 min after injection using Pearl Imager (LI-COR Biosciences). Subsequently, tumors were harvested and imaged *ex vivo* for IR800 fluorescence. The fluorescence intensity of the tumor was quantified using Image J software (National Institutes of Health). To calculate perfusion changes during the ACT, the following formula was used: perfusion change compared to untreated tumor (%) = (fluorescence intensity of tumor with ACT - fluorescence background) / (fluorescence intensity of control tumor - fluorescence background).

### Statistical analysis

Statistical analysis was conducted using GraphPad Prism software. Two-tailed unpaired Student's t-test was utilized to compare 1 variable between two groups. For comparison of multiple groups involving 1 variable, ordinary one-way ANOVA was employed, and for that involving 2 variables, two-way ANOVA was used. For analyzing the tumor growth curves, repeated-measure two-way ANOVA was used. Kaplan-Meier curves were analyzed using log-rank (Mantel-Cox) test was used. Statistical significance was defined as p-values less than 0.05, where 'n' represents the number of replicates. The results are presented as mean values accompanied by the standard error of the mean (SEM).

### Additional materials and methods

Product catalog numbers are summarized in the Supplementary Tables.

## Results

### Requirement for IFN-γR1 expression on non-tumor cells for ACT efficacy

**Figure 1:**
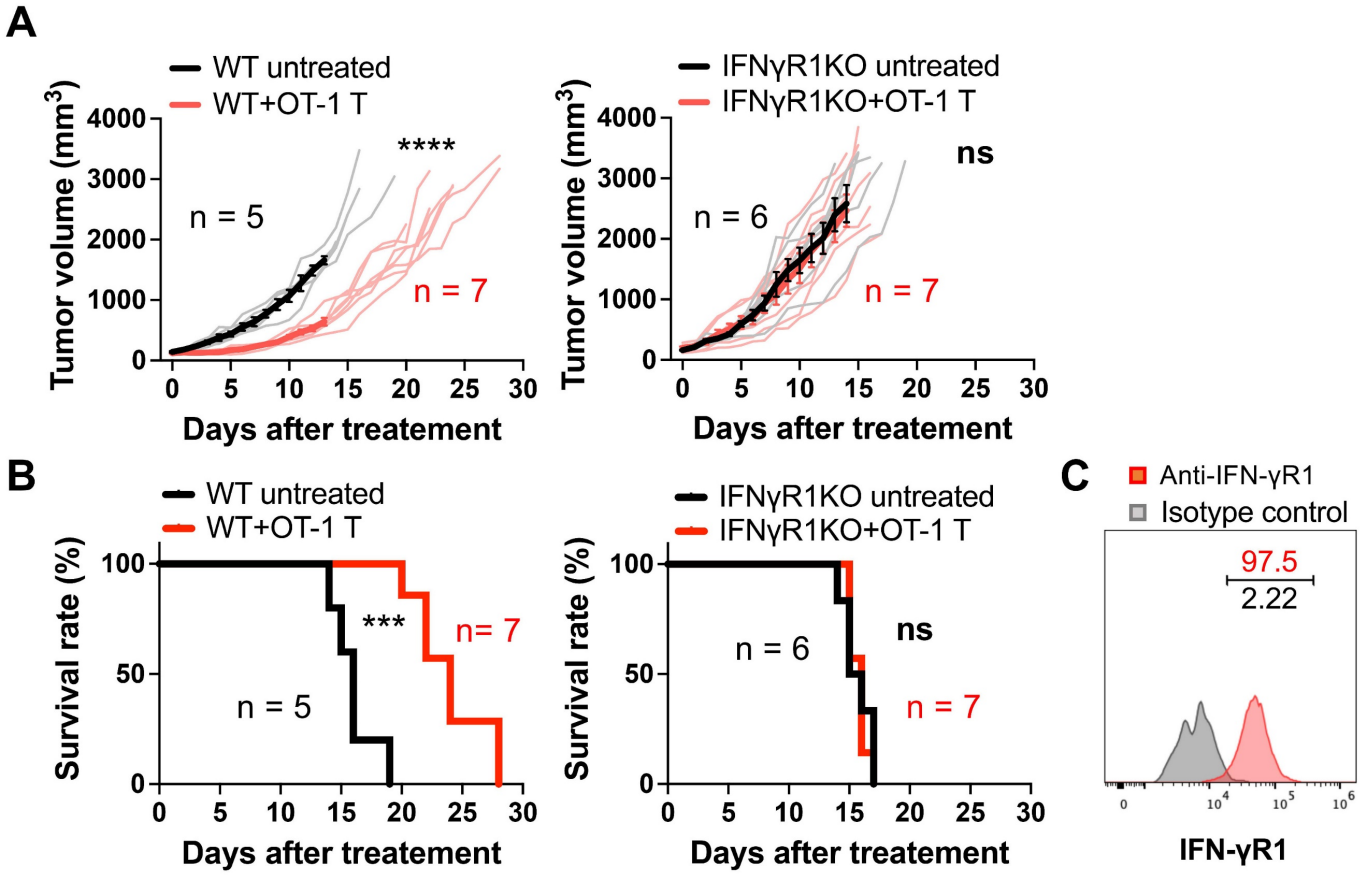
Requirement for IFN-γR1 expression on non-tumor cells for the efficacy of ACT. **A,** ACT targeting OVA antigen suppressed the growth of MCA-205-OVA-GFP fibrosarcoma in WT mice but failed to demonstrate efficacy in IFNγR1KO mice. Thin line: volume of individual tumor, bold line: average tumor volume. ****: P < 0.0001; ns, not significant; analyzed by repeated-measure two-way ANOVA. **B,** ACT prolonged the survival of WT mice bearing MCA-205-OVA-GFP tumors but failed in IFNγR1KO mice. ***: P < 0.001; ns, not significant; analyzed by log-rank (Mantel-Cox) test. **C,** Flow cytometry analysis indicated expression of IFN-γR1 in MCA-205-OVA-GFP tumors *in vivo* (representative data of n = 3).

To examine the contribution of IFN-γ on non-tumor cells in ACT, we performed ACT in WT and IFNγR1KO mice bearing a syngeneic MCA-205-OVA-GFP fibrosarcoma by transferring *ex vivo*-activated OVA-specific OT-1 CD8 T cells. Transfer of three million CD8 T cells successfully suppressed tumor growth in WT mice (Figure 1A), resulting in a significant extension of survival time (Figure 1B). Conversely, in IFNγR1KO mice, the antitumor efficacy was entirely abrogated, with no discernible differences between the treated and untreated groups both in tumor growth and mouse survival (Figure 1A-B). This phenomenon was also observed in another syngeneic tumor model, MOC2-SIINFEKL oral squamous cell carcinoma expressing an OVA peptide (Figure S1A-B). Notably, flow cytometry analysis confirmed the expression of IFN-γR1 in both cancer cell types *in vivo* (Figure 1C and Figure S1C). These data indicate that the efficacy of ACT in suppressing tumor growth requires the expression of IFN-γR1 on non-tumor cells.

### Essential role of endothelial IFN-γR1 expression for antitumor activity of ACT

**Figure 2:**
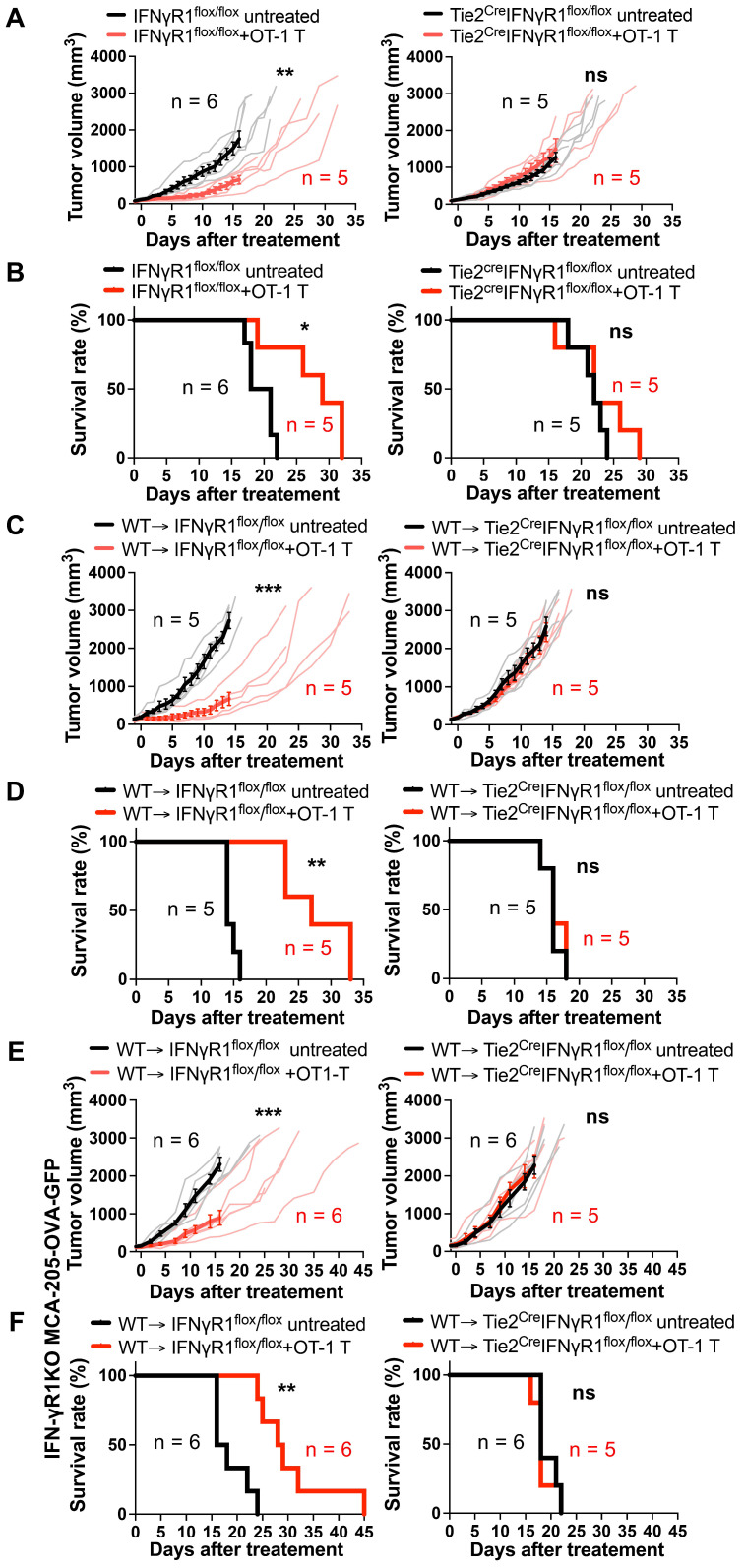
Requirement for IFN-γR1 expression on endothelial cells for the efficacy of ACT. **A,** ACT suppressed MCA-205-OVA-GFP fibrosarcoma growth in control IFNγR1^flox/flox^ mice but failed to demonstrate efficacy in Tie2^cre^IFNγR1^flox/flox^ mice that lack IFN-γR1 expression in both endothelial and some myeloid cells. Thin lines indicate volumes of individual tumors and bold lines indicate average tumor volume. **: P < 0.01, ns: not significant, by repeated-measure two-way ANOVA.** B,** ACT prolonged the survival of IFNγR1^flox/flox^ mice bearing MCA-205-OVA-GFP tumors but not in the Tie2^cre^IFNγR1^flox/flox^ mice. *: P < 0.05, ns: not significant, by log-rank (Mantel-Cox) test. **C,** ACT suppressed MCA-205-OVA-GFP tumor growth in control WT → IFNγR1^flox/flox^ bone marrow chimera mice but not in WT → Tie2^cre^IFNγR1^flox/flox^ bone marrow chimera mice that lack IFN-γR1 expression in endothelial cells, suggesting the requirement of IFNγ action on endothelial cells for the efficacy. Graphs are shown in accordance with those in** A.** ***: P < 0.001, ns: not significant, by repeated-measure two-way ANOVA. **D,** Prolonged survival following ACT was observed in control WT → IFNγR1^flox/flox^ bone marrow chimeras bearing MCA-205-OVA-GFP tumors but was abrogated in WT → Tie2^cre^IFNγR1^flox/flox^ bone marrow chimeras. **: P < 0.01, ns: not significant, by log-rank (Mantel-Cox) test. **E**, ACT suppressed IFN-γR1 deficient MCA-205-OVA-GFP tumor growth in control WT → IFNγR1^flox/flox^ bone marrow chimera mice but not in WT → Tie2^cre^IFNγR1^flox/flox^ bone marrow chimeras that lack IFN-γR1 expression in endothelial cells, suggesting that ACT can be effective without a direct IFN-γ action on the tumor cells. Graphs are shown in accordance with those in** A.** ***: P < 0.001, ns: not significant, by repeated-measure two-way ANOVA. **F**, Prolonged survival following ACT was observed in control WT → IFNγR1^flox/flox^ bone marrow chimeras bearing IFN-γR1KO-MCA-205-OVA-GFP tumors but was abrogated in WT → Tie2^cre^IFNγR1^flox/flox^ bone marrow chimeras. **: P < 0.01, ns: not significant, by log-rank (Mantel-Cox) test.

To discern which non-tumor cells expressing IFN-γR1 are the main target of IFN-γ, we utilized Tie2^cre^IFNγR1^flox/flox^ mice, lacking IFN-γR1 in both endothelial and some of hematopoietic-lineage cells (mainly myeloid cells) 19, 20, alongside their corresponding control IFNγR1^flox/flox^ littermates that express IFN-γR1 on all cells. Infusion of three million OT-1 CD8 T cells significantly suppressed the growth of MCA-205-OVA-GFP tumors in IFNγR1^flox/flox^ mice, extending the survival of these mice compared to untreated controls (Figures 2A-B). In contrast, in Tie2^cre^IFNγR1^flox/flox^ mice, the anti-tumor efficacy of ACT was abrogated, with no discernible difference between the treated group and controls. This suggests that IFN-γR1 expression in either endothelial cells, myeloid-lineage cells, or both, is required for the efficacy of ACT.

Given that Tie2^cre^IFNγR1^flox/flox^ mice lack IFN-γR1 in both endothelial and some myeloid-lineage cells, we constructed bone-marrow chimera mice by infusing WT (Ly5.1) bone marrow cells into lethally irradiated Tie2^cre^IFNγR1^flox/flox^ mice (expressing Ly5.2) (WT → Tie2^cre^IFNγR1^flox/flox^). These chimeras had IFN-γR1 expression on hematopoietic cells restored but lacked it on the endothelial cells. Littermate IFNγR1^flox/flox^ (Ly5.2) mice expressing IFN-γR1 in all cells were used as recipients to generate control chimeras (WT → IFNγR1^flox/flox^). After 8 weeks of transplantation, approximately 95% of peripheral blood mononuclear cells and nearly 99% of CD11b^+^ cells were Ly5.1^+^Ly5.2^-^ cells (Figure S2), indicating a successful reconstitution of hematopoietic cells, including myeloid cells, by the Ly5.1^+^ donor bone marrow-derived cells. These chimera mice were utilized for further investigations determining whether IFN-γR1 expression in endothelial cells and/or myeloid-lineage cells was important for successful ACT. The chimera mice were inoculated with MCA-205-OVA-GFP tumors and OT-1 CD8 T cells were transferred. Tumor growth was suppressed compared to the non-treated group in WT → IFNγR1^flox/flox^ mice (Figures 2C-D) but no suppression was observed in WT → Tie2^Cre^IFNγR1^flox/flox^ mice, suggesting that IFN-γR1 expression in endothelial cells was indispensable for the success of ACT. We observed similar findings in another tumor model, MOC2-SIINFEKL tumor (Figure S3). To further determine if IFN-γ action on endothelial cells alone is sufficient to suppress tumor growth, we performed ACT against IFN-γR1KO-MCA-205-OVA-GFP tumors in the bone marrow chimeras. OT-1 CD8 T cell transfer successfully suppressed growth of the tumor that lack IFN-γR1 in WT → IFN-γR1^flox/flox^ mice, extending the survival, but not in WT → Tie2^Cre^IFNγR1^flox/flox^ mice without IFN-γR1 expression on endothelial cells (Figure 2E-F). These results provide compelling evidence that the efficacy of ACT requires the action of IFN-γ on endothelial cells and that on tumor cells may not be required.

### IFN-γ production within the tumor microenvironment early after ACT

**Figure 3:**
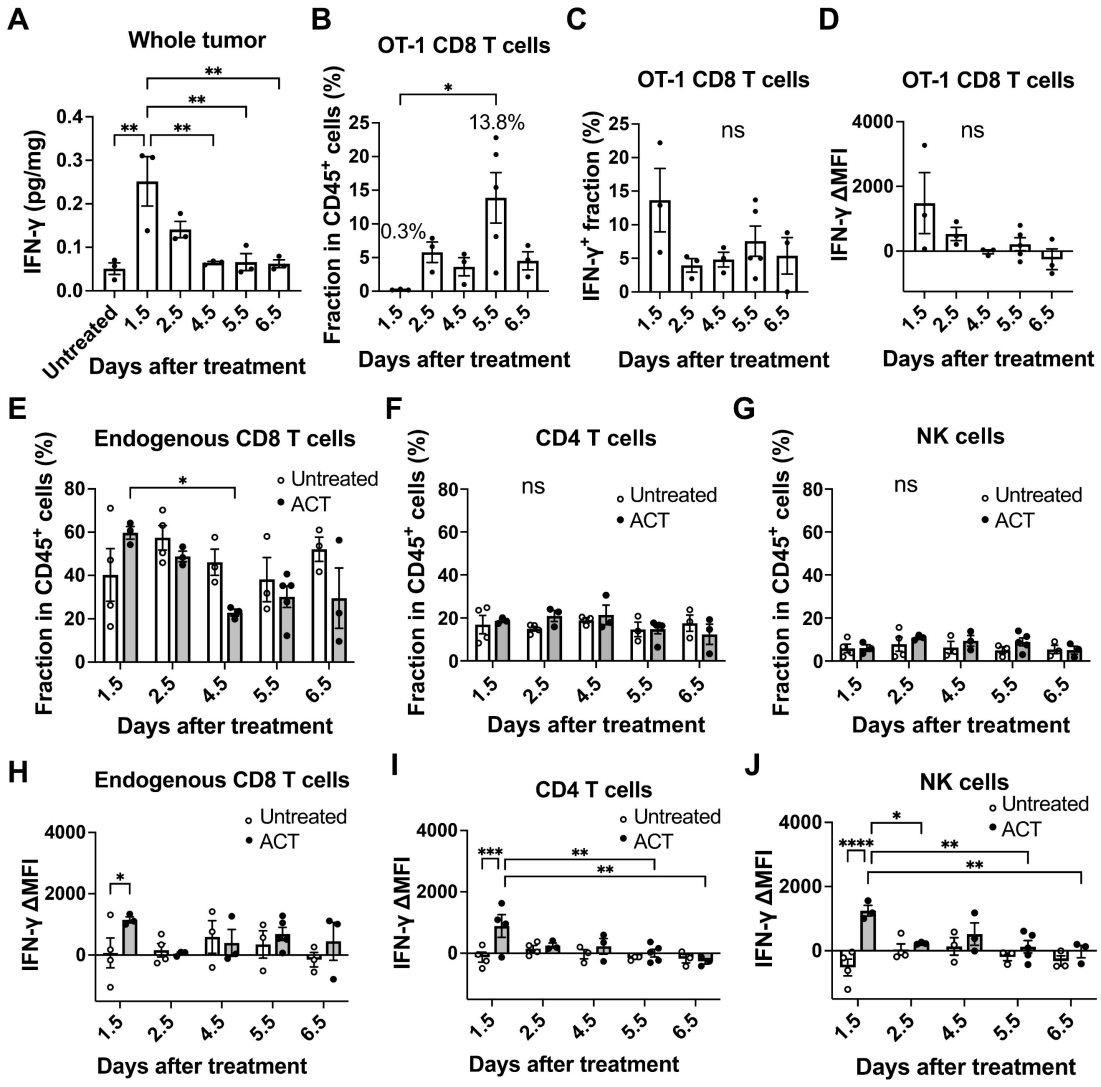
Varying IFN-γ production within the tumor microenvironment during ACT. **A,** ELISA analysis revealed that the IFN-γ level in the whole MCA-205-OVA-GFP tumor tissue showed a peak on Day 1.5 after T cell transfer, decreased until Day 4.5, and plateaued thereafter (n = 3). **: p < 0.01 by one-way ANOVA. **B,** Flow cytometry analysis showed that the fraction of transferred OT-1 CD8 T cells within CD45^+^ hematopoietic cells infiltrating a tumor reached a peak on Day 5.5 after ACT (n = 3-4). *: p < 0.05 by one-way ANOVA. **C and D,** The frequency of IFNγ-producing OT-1 CD8 T cells (**C**) and delta mean fluorescence intensity (ΔMFI) of OT-1 T cells (**D**) infiltrating the tumor were highest on Day 1.5 by flow cytometry analysis (n = 3-4). ns: not significant by one-way ANOVA. **E, F, and G,** Fraction of endogenous CD8 T cells (**E**), CD4 T cells (**F**) and NK cells (**G**) within CD45^+^ hematopoietic cells in the tumor analyzed by flow cytometry (n = 3-5). *: p < 0.05, **: p < 0.01, ns: not significant, by two-way ANOVA. **H, I, and J,** IFN-γ ΔMFI was further analyzed for endogenous CD8 T cells (expressing Thy1.2) (H), CD4 T cells (I) and NK cells (J) infiltrating untreated tumors and tumors treated by transfer of OT-1 CD8 T cells (expressing Thy1.1) at various time points (n = 3-5). *: p < 0.05, **: p < 0.01, by two-way ANOVA.

We next investigated the production of IFN-γ within the tumor microenvironment during ACT. We measured IFN-γ concentrations in the tumor lysates by enzyme-linked immunosorbent assay (ELISA) and found that the IFN-γ level in the whole tumor tissue reached its peak at Day 1.5 after T cell transfer, reaching a concentration of 0.25 pg/mg of tissue and decreasing thereafter (Figure 3A). Subsequently, we employed flow cytometry to examine IFN-γ production by OT-1 CD8 T cells and endogenous CD8 T, CD4 T and NK cells infiltrating the tumor (Figure S4A). OT-1 CD8 T cells started to be detectable in the tumor at Day 1.5 and their fraction in CD45^+^ hematopoietic cells reached the peak at Day 5.5 (13.8%) after transfer (Figure 3B). However, the frequency of OT-1 CD8 T cells producing IFN-γ (Figure 3C) and the level of IFN-γ production (delta mean fluorescence intensity, ΔMFI, Figure 3D, Figure S4B) were highest at Day 1.5. The fraction of endogenous CD8 T cells and CD4 T cells ranged from approximately 20-60% and 10-20% of CD45^+^ cells, respectively (Figure 3E-F). The fraction of endogenous CD8 T cells decreased at Day 4.5, which might have resulted from influx of phagocytes such as monocytes when cancer cell started to die. Endogenous NK cells remained low among CD45^+^ cells (Figure 3G). IFN-γ production (ΔMFI) in endogenous CD8 T cells (Figure 3H), CD4 T cells (Figure 3I) and NK cells (Figure 3J) significantly increased in the ACT group compared to the untreated group on Day 1.5, but no significant difference was observed between the two groups on Days 2.5-6.5 (Figure 3H-J, Figure S4B). The OT-1 CD8 T cells within the tumor accounted for only 0.3% of CD45^+^ cells at Day 1.5. Despite their low abundance, they were activated and produced high amounts of IFN-γ (Figure 3C-D), which likely activated endogenous tumor-infiltrating lymphocytes. The high levels of IFN-γ produced by OT-1 CD8 T cells, combined with the contribution from endogenous T and NK cells, led to the highest IFN-γ concentrations being observed at Day 1.5 after transfer. In contrast, the highest accumulation of OT-1 CD8 T cells (13.8%) was observed on Day 5.5, but these T cells were no longer producing IFN-γ (Figure 3D). Of note, when naïve OT-1 CD8 T cells with no IFN-γ production were used for ACT against MCA-205-OVA-GFP tumor, they were unable to suppress tumor growth even with increasing doses of cells (Figure S4C).

### Early IFN-γ targeting of tumor vascular endothelial cells induced by a limited number of high IFN-γ-producing transferred tumor-specific CD8 T cells

**Figure 4:**
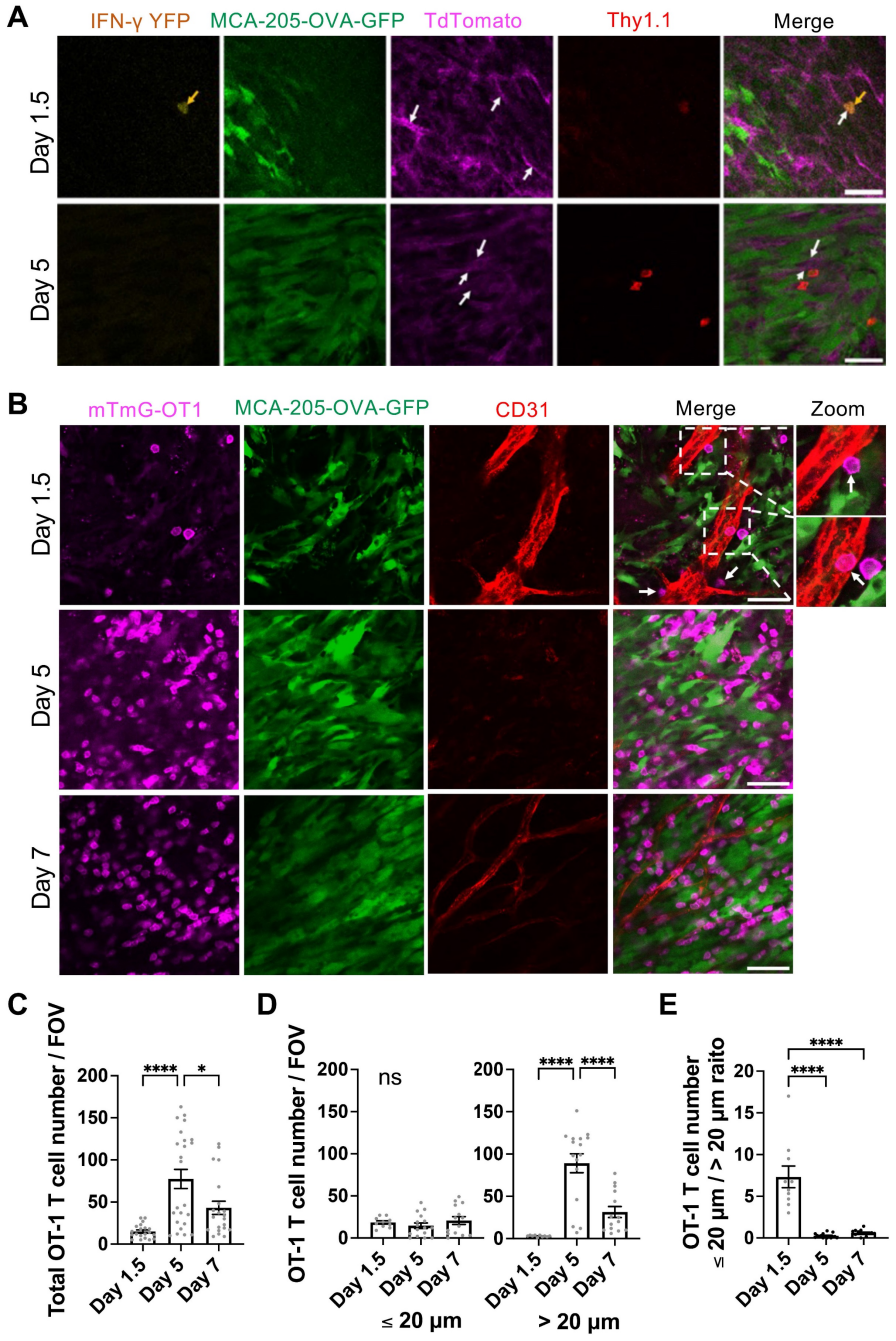
Interaction of IFN-γ producing transferred tumor-specific CD8 T cells with tumor vascular endothelial cells early after ACT. **A,** Intravital images demonstrated presence of transferred IFN-γ-IRES-eYFP-OT-1 CD8 T cells around the location of tumor blood vessels on Day 1.5 and Day 5 after ACT. Green fluorescent protein (GFP) fluorescence represents MCA-205-OVA-GFP tumor cells, while Tdtomato fluorescence (magenda) of mTmG mice indicates the structure of blood vessels (white arrows). IFN-γ-IRES-eYFP-OT-1 CD8 T cells (yellow arrows) derived from GREAT-OT1-Thy1.1 mice were labeled by intravenously injected APC-conjugated anti-Thy1.1 antibody (red) and exhibited YFP fluorescence signal (yellow) on Day 1.5 indicating IFN-γ production. Scale bar: 50 µm. Representative images of n = 3. **B**. Representative intravital images of mTmG-OT-1 CD8 T cells expressing Tdtomato (magenda) transferred to MCA-205-OVA-GFP tumor (green)-bearing wild type mice. Tumor vessels were labeled by intravenously injected Alexa Fluor 647-conjugated anti-CD31 antibody (red). mTmG-OT-1 T cells were detected inside and near tumor vessels on Day 1.5. On Day 5, while mTmG-OT-1 T cells infiltrated in the tumor bed, vessels were regressed. On Day 7, recovery of the tumor vessels was observed. A 2-fold magnified view of the dashed boxed area on Day 1.5 is provided on the far right. White arrows indicate mTmG-OT-1 CD8 T cells attached to the endothelial cells. Scale bar: 50 µm. Representative images of n = 4 (5 FOV each), for Day 1.5, n = 6 (4 FOV each) for Day 5 and n = 3 (7 FOV each), for Day 7. Also see movies S1-3. **C**. Number of OT-1 T cells found per FOV of the images obtained in B. *: p < 0.05, ****: p < 0.0001, by one-way ANOVA. **D**. Number of OT-1 T cells found within 20 µm (left) and greater than 20 µm (right) of tumor vessels per FOV of the images obtained in B. Quantitation was performed on n = 2 (5 FOV each) on Day 1.5, n = 4 (4 FOV each) on Day 5 and n = 2 (7 FOV each) on Day 7. ****: p < 0.0001, ns: not significant, by one-way ANOVA. **E**. Ratio of OT-1 T cells located within 20 µm and greater than 20 µm of vessels, analyzed in **D**, are shown. ****: p < 0.0001 by one-way ANOVA.

Because IFN-γ concentration in the tumor peaked as early as Day 1.5 after T cell transfer, we performed intravital microscopy to examine the kinetics of OT-1 CD8 T cell infiltration into the tumor, with possible interaction with the endothelial cells during vascular extravasation. CD8 T cells derived from GREAT-OT1-Thy1.1 mice (IFN-γ-IRES-eYFP-OT-1 T cells), which exhibit YFP fluorescence signal upon IFN-γ production, were activated *ex vivo* and transferred to mTmG mice (expressing Thy1.2) bearing MCA-205-OVA-GFP tumor. TdTomato fluorescence of mTmG mice allows visualization of blood vessels. Transferred IFN-γ-IRES-eYFP-OT-1 CD8 T cells were labeled *in vivo* by intravenously injected APC-conjugated anti-Thy1.1 antibody. Intravital imaging of the tumor on Day 1.5 after T cell transfer revealed a very limited number of IFN-γ-IRES-eYFP-OT-1 CD8 T cells (Figure 4A). These cells displayed strong YFP fluorescence, suggesting high IFN-γ production, and were localized in or near blood vessels. In contrast, on Day 5, IFN-γ-IRES-eYFP-OT-1 CD8 T cells in the tumor tissue exhibited no detectable YFP fluorescence signal, indicating minimal IFN-γ production (Figure 4A). This IFN-γ production pattern was consistent with flow cytometry data (Figure 3).

We further used mTmG-OT-1 CD8 T cells to treat MCA-205-OVA-GFP tumor grown in WT mice. APC-conjugated anti-CD31 antibody was infused to label the blood vessels. Transferred mTmG-OT-1 T cells (Figure 4B and Movie S1) were observed either inside the blood vessel, traversing through endothelial cells, or in the perivascular regions (within a 20 µm distance) on Day 1.5, indicating close proximity with endothelial cells. In contrast, on Days 5 and 7, a higher number of mTmG-OT-1 cells were found within the tumor microenvironment, located > 20 µm distant from tumor vessels (Figure 4B-E and Movie S2-3). Interestingly, tumor vessels were barely detectable on Day 5 and only narrowed vessels were scarcely seen on Day 7.

**Figure 5:**
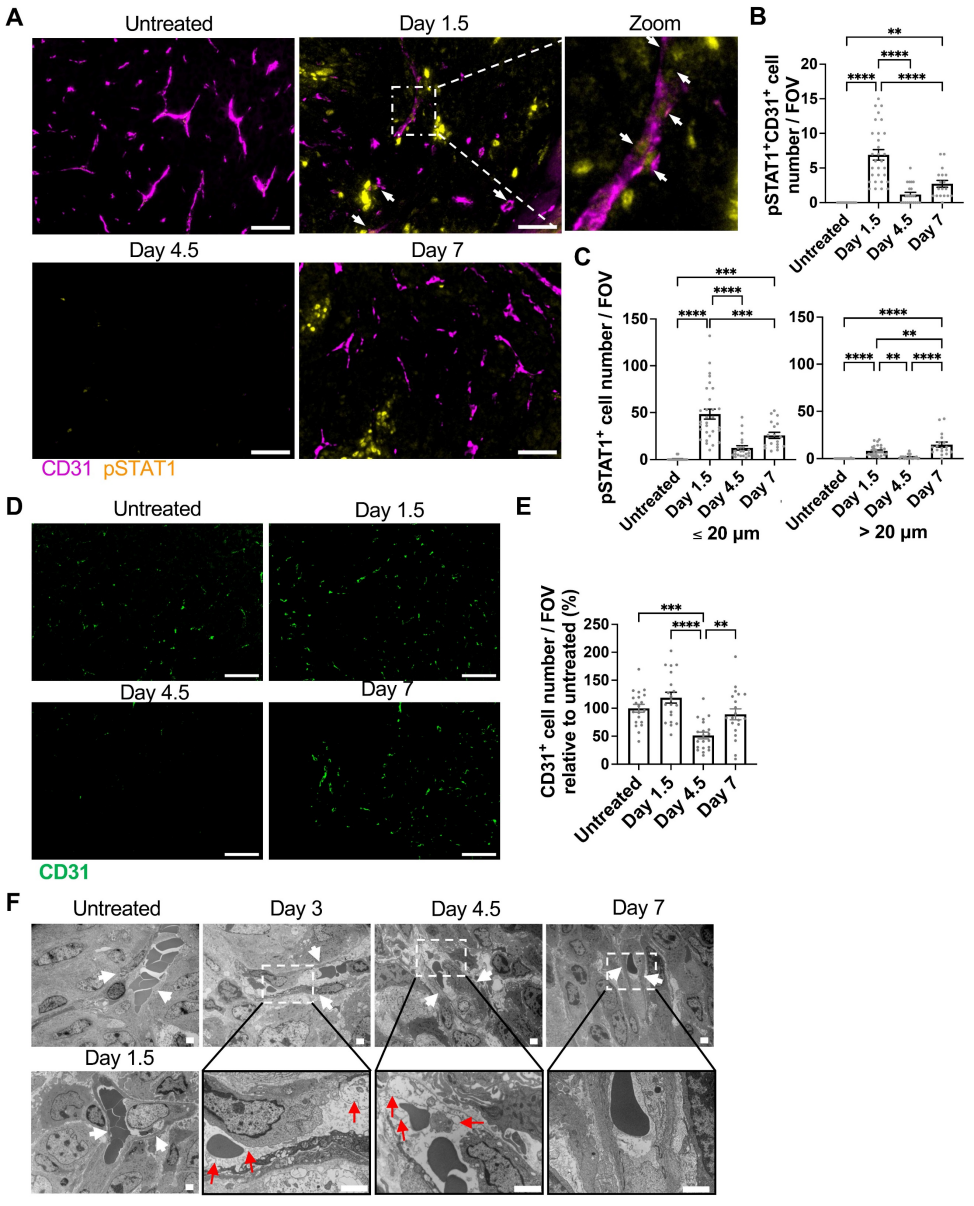
Induction of STAT1 signaling in tumor vessel endothelial cells and vessel regression by ACT. **A,** Representative immunofluorescence images of CD31 and pSTAT1 staining in MCA-205-OVA-GFP tumor tissue section in the untreated group and at Day 1.5, Day 4.5, and Day 7 after ACT (n = 2 for untreated and Days 4.5 and 7, n = 3 for Day 1.5), White arrows indicate pSTAT1^+^CD31^+^ cells. A 4-fold magnified view of boxed area on Day 1.5 is shown far right. Scale bar: 50 µm. **B,** pSTAT1^+^CD31^+^ cell number per FOV imaged in **D** at different time points after ACT is summarized. Each symbol represents one individual FOV. For untreated tumor, 11 FOVs were analyzed, For Days 1.5, 4.5 and 7, 10, 11, and 9 FOVs, respectively, were analyzed. **, P < 0.01; ****, P < 0.0001, by one-way ANOVA. **C,** pSTAT1^+^ cell number per FOV were quantitated by the distance from the closest tumor vessel using the same FOVs analyzed in E. **, P < 0.01; ***, P < 0.001; ****, P < 0.0001, by one-way ANOVA. **D**, Representative immunofluorescence images of MCA-205-OVA-GFP tumor tissue sections in the untreated tumor and tumors at Day 1.5, Day 4.5, and Day 7 after ACT (n = 2 tumors, 20 FOVs per group were analyzed), Scale bar: 50 µm. **E**, Quantitation of the images acquired in **A** shown as CD31^+^ cell number per FOV relative to that of the untreated tumors (2 tumors, total of 20 FOVs per time point). **, p < 0.01, ***, p < 0.001, and ****, p < 0.0001, by one-way ANOVA. **F,** Transmission electron microscopy images depicting degeneration of endothelial cells (white arrows indicate blood vessels; red arrows indicate obscured intracellular organelles with or without cytoplasmic vacuolation) at Days 3-4.5 after ACT on MCA-205-OVA-GFP tumors. The vessels started to recover at Day 7 (n = 2). Scale bar, 2 μm.

Consequently, we investigated the distribution of cells receiving IFN-γ in the tumor microenvironment by examining the phosphorylation of STAT1, a downstream molecule of the IFN-γ signaling, on tyrosine-701 (pSTAT1) 21 at various time points following T cell transfer. We employed multiplex immunofluorescence staining of endothelial cells (labeled by CD31) and pSTAT1. As illustrated in the low magnification views of the tumor (Figure S5A) with quantification (Figures S5B-C), both the pSTAT1 expression level and the number of pSTAT1 positive cells observed within a field of view (FOV) in the tumor were highest at Day 1.5 after T cell transfer compared to Days 4.5 and 7 or the untreated tumor. By analyzing the colocalization of pSTAT1 signal and CD31^+^ endothelial cell staining, we observed pSTAT1^+^ positive tumor vascular endothelial cells (pSTAT1^+^CD31^+^ cells) in the tumor, peaking on Day 1.5 (Figure 5A-B). Furthermore, the number of pSTAT1^+^ cells within 20 µm of tumor vessels also peaked at Day 1.5, while pSTAT1^+^ cells detected further away from the tumor blood vessels (> 20 µm) was comparatively diminished but peaked at Day 7 (Figure 5C). These findings indicate that IFN-γ from tumor-specific CD8 T cells, possibly combined with that from endogenous lymphocytes, activates STAT1 signaling in the tumor endothelial cells as early as Day 1.5 following T cell transfer, whereas IFN-γ effects are limited on the cells more distant from the vessels even at later time points. Of note, IFN-γ levels in the serum remained minimal or under the detection limit throughout the observation period (Figure S6A) and no pSTAT1 was detected in the liver and spleen (Figure S6B), indicating that although the transferred IFN-γ-producing CD8 T cells circulated throughout body, there was no IFN-γ effect on “normal” organs.

Multiplex ELISA was performed on the whole tumor at different time points after T cell transfer to investigate changes in cytokines and chemokines within the tumor microenvironment following ACT. As shown in Figure S7, IFN-γ-inducible chemokines CXCL9 and CXCL10, as well as CCL5, CCL11, and granulocyte-macrophage colony-stimulating factor (GM-CSF) and tumor necrosis factor alpha (TNFα), exhibited a significant increase at Day 1.5 compared to the untreated control, while interleukin (IL)-12p70 significantly decreased. CXCL2 also increased on Days 1.5 and 2.5 and IL-1α on Day 1.5, although the increases were not significant compared to the untreated control. The levels of IL-2 did not significantly change during the observation. Moreover, VEGF levels showed an increase on Day 6.5, although not statistically significant likely due to the considerable variations among tumors. The data indicated that the delivery of tumor-specific CD8 T cells and IFN-γ targeting induced significant changes in the profile of cytokines and chemokines within the tumor microenvironment.

### Induction of tumor vessel regression, tumor hypoxia, and necrosis by ACT

After confirming the activation of STAT1 signaling in tumor endothelial cells after ACT, we further analyzed endothelial cells by CD31 immunofluorescence imaging of the harvested tumors. The CD31^+^ endothelial cell density significantly decreased at Day 4.5 compared to the untreated group and other time points (Figure 5D-E). Further, we performed *in vivo* endothelial cell labeling by infusing an APC-anti-CD31 antibody at different time points after ACT, before harvesting the tumor. This method enables differentiation of endothelial cells associated with perfused tumor vessels from perfusion-impaired tumor blood vessels that remain unlabeled. This “functional” CD31 staining demonstrated a significant decrease in perfused-vessel endothelial cells at Day 4.5 (Figure S8). Using electron microscopy, we observed endothelial cell degeneration characterized by obscured intracellular organelles with or without cytoplasmic vacuolation as early as Days 3-4.5 after T cell transfer and its recovery at Day 7 (Figure 5F).

**Figure 6:**
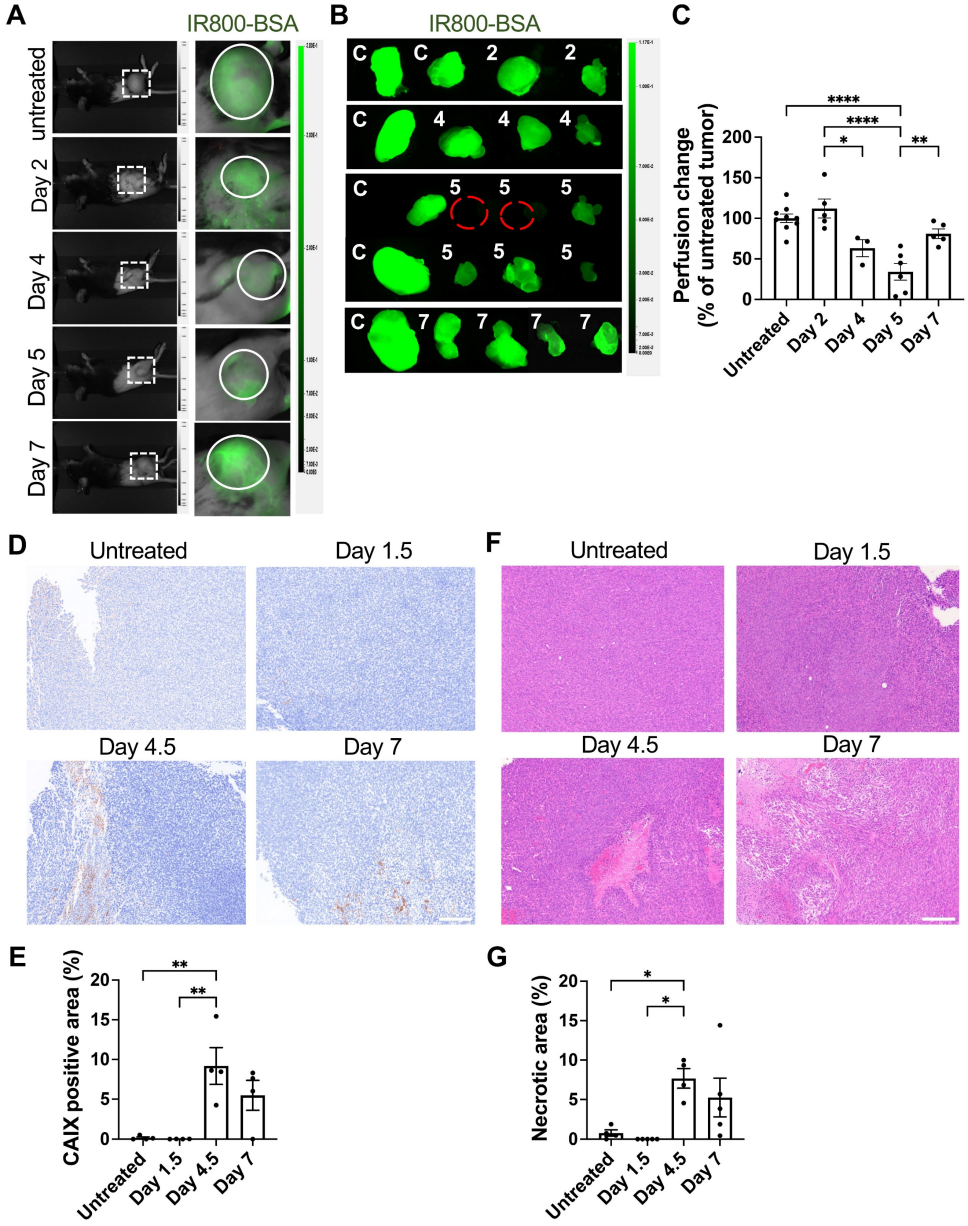
Induction of impaired tumor vessel perfusion, tumor hypoxia, and necrosis after ACT. **A,** Whole-body imaging at 20 mins after intravenous injection of 50 μg IR800-conjugated albumin (IR800-BSA) in MCA-205-OVA-GFP tumor-bearing mice untreated or at indicated time points after ACT. Visual light (left) and fluorescent images visualizing IR800-BSA (right). Representative images (untreated: n = 9, Days 2 and 7: n = 5, Day 4: n = 3, Day 5: n = 6), **B**, Representative* ex vivo* fluorescence images of the tumors harvested after the imaging performed in **A** (n = 3-7). Untreated tumor (C) and tumors from ACT-treated mice (numbers indicate numbers of days after ACT). **C**, Fluorescent intensity of the treated tumors in **B** were quantitated and normalized to those in untreated tumors in each experiment (n = 3-7). Each symbol represents individual tumor. *, P < 0.05; **, P < 0.01; ****, P < 0.0001, by one-way ANOVA. D**,** Representative IHC images of carbonic anhydrase IX (CAIX) staining in MCA-205-OVA-GFP tumor tissue sections in the untreated group and after 1.5 days, 4.5 days, and 7 days of ACT (n = 2 tumors). Scale bar: 200 µm. **E,** Quantitation of CAIX-positive tumor area/total tumor area ratio in the whole-tumor images obtained at four-fold magnification (two tumors per group, 2 whole-tumor section each, total of 4 sections per group). **, P < 0.01, by one-way ANOVA. **F,** Representative H&E staining images of MCA-205-OVA-GFP tumor tissue sections in the untreated group and after 1.5 days, 4.5 days, and 7 days of treatment (untreated: n = 4, Days 1.5 and 7: n = 5, Days 4.5: n = 4 tumors). Scale bar: 200 µm. **G,** Quantitation of necrotic tumor area/total tumor area ratio in the whole-tumor images obtained at four-fold magnification (untreated: n = 4, Days 1.5 and 7: n = 5, Days 4.5: n = 4). *, P < 0.05, by one-way ANOVA.

**Figure 7:**
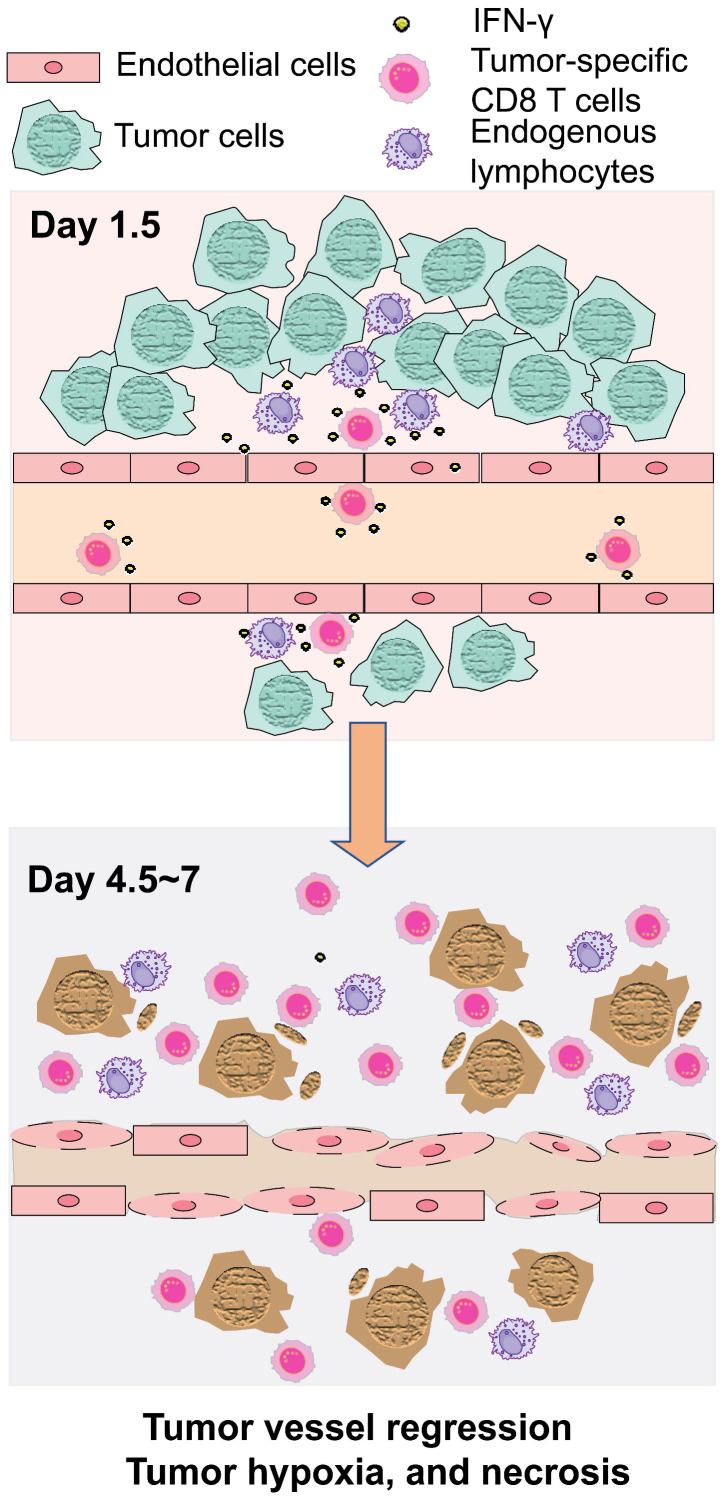
Schematic illustration of tumor growth suppression in adoptive T cell therapy through IFN-γ targeting of tumor vascular endothelial cells.

Examination of tumor vessel perfusion by infusion of NIR fluorescent IR800-labeled albumin revealed a significant reduction of *in vivo* tumor blood perfusion on Days 4 and 5 following T cell transfer (Figure 6A-C). Associated with the regression of blood perfusion, expression of carbonic anhydrase IX (CAIX), an ischemic/hypoxic marker, increased on Day 4.5 and Day 7 (Figure 6D), indicating intratumoral hypoxia. The distribution of CAIX positive hypoxic areas was heterogenous on Days 4.5 and 7, but quantitation revealed significant increase in the CAIX positive area relative to the whole tumor area (Figure 6E). Similarly, H&E staining revealed significant increase in tumor tissue necrosis 4-7 days after T cell transfer (Figure 6F-G). Altogether, these data demonstrate that IFN-γ produced by exogenous T cells, together with IFN-γ released from endogenous lymphocytes residing perivascular area, acted upon endothelial cells to cause tumor vessel regression, leading to hypoxia and necrosis and tumor growth suppression, as illustrated in Figure 7.

## Discussion

IFN-γ is considered a crucial mediator of tumor response in ACT. It has been reported that the effects of IFN-γ are both dose and spatiotemporally dependent; low-dose IFN-γ treatment may provoke metastases, while high-dose IFN-γ leads to tumor regression 17, 18. Recent reports utilizing mosaic models of antigen loss in tumors and intravital imaging have shown the far-reaching effects of IFN-γ secreted by CD8 T cells on tumor cells. These studies demonstrate that the spatial distribution of diffusible IFN-γ can significantly influence tumor growth 22, 23. The concentration and location of IFN-γ within the tumor microenvironment may therefore determine its functional outcome. A comprehensive understanding of the spatiotemporal dynamics of IFN-γ effects within the tumor microenvironment is important in improving response.

Using *ex vivo*-activated OVA-specific OT-1 CD8 T cells in OVA- or OVA-peptide-expressing syngeneic mouse tumor models, we demonstrated that IFN-γ produced by a limited number of transferred CD8 T cells targeted tumor vascular endothelial cells early after ACT (Day 1.5), thus triggering profound antitumor effects. Transferred CD8 T cells also induced IFN-γ production in pre-existing tumor-infiltrating lymphocytes, likely localized near the tumor vessels, which might also have contributed to the IFN-γ effect on the vascular endothelial cells. Phosphorylation of STAT1 in the tumor vessel endothelial cells, as well as on some cells around the vessels, were observed at Day 1.5, which is approximately the time infused OT-1 CD8 T cells begin to home to OVA-expressing tumors 24. Activation of IFN-γ/STAT1 signaling is reported to disrupt endothelial barrier integrity 25-27. In our study, IFN-γ led to endothelial cell regression, reduced perfusion, tumor hypoxia, and necrosis (Day 4.5-7 post-ACT), ultimately resulting in tumor regression. The hypoxic and necrotic areas were distributed in a heterogeneous manner throughout the tumor and were likely downstream of endothelial cells affected by IFN-γ. Our group previously observed a similar IFN-γ-mediated regression of endothelial cells, leading to tumor hypoxia and regression under physiological conditions in regulatory T cell (Treg)-targeted near-infrared photoimmunotherapy 19. Depletion of intratumoral Tregs induces synchronized activation of tumor-infiltrating CD8 T and NK cells, predominantly residing in perivascular regions, and results in rapid IFN-γ production targeting vascular endothelial cells 19. Although, in ACT, CD8 T cells infused systemically were producing high concentrations of IFN-γ, we observed no pSTAT1 expression in normal organs. It is possible that the interaction time between tumor-specific T cells and tumor vascular endothelial cells is longer than that with endothelial cells in normal organs, due to complex mechanisms; involving chemokine-mediated recruitment and augmented adhesion of activated T cells to inflamed tumor vascular endothelial cells, presence of tumor-associated high endothelial venules that enhance recruitment and transendothelial extravasation of T cells, and tumor-antigen expression by endothelial cells in the context of MHC, which can be recognized by tumor-specific T cells 28-30. The early encounter of *ex vivo* activated CD8 T cells with tumor vasculature via IFN-γ, prior to the T cells' exposure to tumor antigens expressed on the cancer cells, may provide clues to solving the problem of antigen escape, a problem that hinders the efficacy of ACT.

At Day 1.5, peak IFN-γ production altered the cytokine and chemokine profiles in the tumor microenvironment, collaborating with IFN-γ/pSTAT1 signaling to induce tumor suppression following ACT 31. Elevated levels of CXCL9, CXCL10, CCL5, CCL11, GM-CSF and TNFα, along with a decline in IL-12p70, were observed. CXCL2 and IL-1α also showed an increase on Days 1.5 and 2.5 and Day1.5, respectively, although these changes were not significant. CXCL9 and CXCL10 are well known IFN-γ-inducible chemokines produced by endothelial cells, monocytes/macrophages and fibroblasts in the tumor microenvironment and prevent tumor angiogenesis by blocking endothelial cell proliferation, inducing tumor necrosis 32-35. CCL5 and CCL11 are in endothelial cells in response to IFN-γ. GM-CSF, produced by a variety of cell types including endothelial cells and other stromal cells, activated T cells and macrophages, along with IFN-γ, demonstrate a strong association with antitumor efficacy 36, 37. IFN-γ also plays a key role in IL-12 production by dendritic cells and myeloid cells and IL-12, in turn, amplifies IFN-γ production 38, 39. In addition, CXCL9 and CXCL10 regulate the antitumor effects of IL-12 40. Interestingly, we observed a decrease in IL-12p70 levels upon IFN-γ activation. This decline may be attributed to the involvement of TNF in the inhibition of IFN-γ-induced IL-12 production 41. In addition, IL-1 is also reported to activate antigen-specific CD8 T cells that are granzyme B positive and produce IFN-γ 42. The intricate crosstalk between these cytokines and chemokines suggests the involvement of complex multiple feedback loops. In our study, IFN-γ seemed to orchestrate the interplay of these cytokines and chemokines in a concentration-dependent manner to facilitate tumor regression.

At Day 6.5 to 7, we observed the restoration of tumor blood vessels evidenced by increased vessel density and perfusion compared to Day 4.5 to 5, along with normalization of endothelial cells on Day 7 observed by transmission electron microscopy. Additionally, VEGF increase was observed in some mice on Day 7, although not statistically significant, which could promote the formation of new blood vessels 43, 44. Combining ACT with antiangiogenic therapy, such as anti-VEGF therapy, has been proposed to enhance treatment efficacy 45, 46, while an antiangiogenic therapy alone has shown limited efficacy in patients. An anti-VEGF therapy increases the CTLs extravasation in tumor microenvironment 47; reduce T cell apoptosis induced by Fas/FasL signaling 48, and inhibit CD8 T cell exhaustion mediated by the PD-1/PDL-1 interaction 49, 50. However, the optimal timing for initiating antiangiogenic therapy in combination with ACT remains a subject of investigation. Our study suggests that Day 4.5 to 5 after ACT might be a suitable time window to commence antiangiogenic therapy and encourages further investigation.

In addition to tumor vascular endothelial cells, tumor-associated lymphatic endothelial cells can also be targeted by IFN-γ produced by T cells. In ACT against VEGF-C-overexpressing OVA^+^ melanoma, which exhibits augmented tumor-associated lymphatic vessel development, the transfer of OT-1 CD8 T cells induce apoptosis in lymphatic endothelial cells 4-5 days later 51. This apoptosis is triggered by the recognition of tumor antigens cross-presented on lymphatic endothelial cells by OT-1 T cells and is dependent on IFN-γ signaling in lymphatic endothelial cells. Consequently, the reduced lymphatic flow from the tumor diminishes cancer cell metastasis to lymph nodes 51. While further investigation is required to determine whether these findings hold in tumor microenvironments with physiological levels of VEGF-C, it is conceivable that regression of tumor vasculature following ACT, as observed in our study, contributes to a decrease in cancer metastasis via blood circulation.

This study has several limitations. The levels of IFN-γ in the tumor microenvironment declined from Day 2.5, suggesting that the anti-tumor function of transferred CD8 T cells may have been relatively short-lived. This could be caused by immune suppressors in the tumor such as Tregs and myeloid-derived suppressor cells. As the OT-1 CD8 T cells were activated *in vitro* before transfer, it is possible that these T cells started to exhaust after further stimulation with tumor-expressed antigens. Additionally, in clinical settings, patients often undergo prior conditioning before cell transfer to ablate endogenous lymphocytes and thus increase T cell tumor infiltration and enhance the survival and proliferation of transferred T cells. In our study, the mice did not undergo a conditioning regimen prior to ACT. This may have reduced the reserve of cytokines required to maintain activation status of the transferred T cells. More comprehensive analyses of transferred CD8 T cells after their homing to the tumor could provide useful information to improve ACT efficacy.

In conclusion, our study highlights the critical role of CD8 T cell-derived IFN-γ targeting endothelial cells in tumor suppression during ACT and elucidates the spatiotemporal dynamics of IFN-γ production *in vivo*. Remarkably, a small fraction of exogenous tumor-specific CD8 T cells producing high concentrations of IFN-γ initiated the process of tumor regression at early time points, preceding any direct effects on tumor cells. Our findings could provide the basis for a strategy for overcoming the challenge of tumor antigen escape observed in clinical ACT.

## Supplementary Material

Supplementary materials and methods, figures and tables, movie legends.

Supplementary movie 1.

Supplementary movie 2.

Supplementary movie 3.

## Abbreviations

ACT: adoptive T cell therapy
APC: allophycocyanin
CAIX: carbonic anhydrase IX
CAR-T: chimeric antigen receptor-T
CRISPR: clustered regularly interspaced short palindromic repeats
DAPI: 4',6-aiamidino-2-phenylindole
ELISA: enzyme-linked immunosorbent assay
FFPE: formalin-fixed paraffin-embedded
GM-CSF: granulocyte-macrophage colony-stimulating factor
H&E: hematoxylin and eosin
IFN-γ: interferon-gamma
IFNγR1: Interferon-γ receptor 1
IL: interleukin
ΔMFI: delta mean fluorescence intensity
MHC-I: major histocompatibility complex class I
OVA: ovalbumin
PBS: phosphate-buffered saline
pSTAT1: phosphorylation of STAT1
SEM: standard error of the mean
TCR-T: T cell receptor (TCR) transduced T
TEM: transmission electron microscopy
TILs: tumor-infiltrating lymphocytes
TNFα: tumor necrosis factor alpha
WT: wild-type
