# Supplementary material for: Tumor growth suppression in adoptive T cell therapy via IFN-γ targeting of tumor vascular endothelial cells
Source: Theranostics. 2024 Oct 21;14(18):6897–912. doi: 10.7150/thno.101107 (PMC11610145; doi:10.7150/thno.101107)
Supplement: Supplementary file 1 — Supplementary materials and methods, figures and tables, movie legends. [file thnov14p6897s1.pdf]

Supplementary information for

Tumor growth suppression in adoptive T cell therapy via IFN-γ targeting of tumor vascular endothelial cells

Qiaoya Lin[1], Colleen P. Olkowski[1], Peter L. Choyke[1], Noriko Sato[1,*]

1. Molecular Imaging Branch, Center for Cancer Research, National Cancer Institute, National Institutes of Health, Bethesda, Maryland.

***Correspondence:**

Noriko Sato, MD, PhD. Molecular Imaging Branch, Center for Cancer Research, National Cancer Institute, National Institutes of Health, Bethesda, MD 20892

Phone: 1+240-858-3079; Fax: 1+240-541-4526

Email: saton@mail.nih.gov

Supplementary materials and methods

The short-term *in situ* intravital imaging

After disinfecting the mouse using betadine solution and 70% ethanol, the tumor area was delineated with a marker pen. The epidermis was carefully incised using forceps and scissors. Sterile saline was applied to the tumor area to prevent tissue desiccation. Subsequently, the mouse was positioned in either the right or left decubitus position, depending on the tumor inoculation site, within a custom-designed chamber. To stabilize the xenograft tumor, surgical tape was applied to the silicone bed, and a metal frame was securely affixed using homemade nuts. The mice were then prepared for intravital imaging studies, with a maximum imaging duration of 6 h. Following intravital imaging, mice were promptly euthanized via cervical dislocation while under anesthesia.

Supplementary tables

Table S1: Antibodies used in the study.

**Antibodies for flow cytometry**	**Source**	**Clone**	**Catalog #**
Anti-mouse CD119 (IFNγR1) antibody, PE	Thermo Fisher Scientific	2E2	2069166
Armenian Hamster IgG isotype control antibody, PE	Thermo Fisher Scientific	eBio299Arm	12-4888-83
Anti-mouse CD45.1 (Ly5.1) antibody, PE	BioLegend	A20	110708
Anti-mouse CD45.2 (Ly5.2) antibody, APC	Thermo Fisher Scientific	104	17-0454-82
Anti-mouse CD8 antibody, Brilliant Violet 650	Thermo Fisher Scientific	53-6.7	100742
Anti-mouse NK1.1 antibody, PE	Thermo Fisher Scientific	PK136	12-5941-83
Anti-mouse IFNγ antibody, APC	BioLegend	XMG1.2	505810
Rat IgG1, k isotype control antibody, APC	BioLegend	RTK2071	400412
Anti-mouse-CD90.1 (Thy1.1) antibody, PE-Cy7	Thermo Fisher Scientific	HIS51	25-0900-82
Anti-mouse-CD90.2 (Thy1.2) antibody, eFluor 450	Thermo Fisher Scientific	53-2.1	48-0920-82
Anti-mouse CD11b antibody, FITC	PharMingen/BD Biosciences	M1/70	017140
Anti-mouse CD16/32 antibody	Thermo Fisher Scientific	93	4289662
**Antibodies for *in vivo* infusion**	**Source**	**Clone**	**Catalog #**
Anti-mouse CD90.1 (Thy1.1) antibody, APC	Thermo Fisher Scientific	HIS51	17-0900-82
Anti-mouse CD31 antibody, Alexa Fluor 647	BioLegend	390	102416
Anti-mouse CD31 antibody, APC	Thermo Fisher Scientific	390	17-0311-80
**Antibodies for histology**	**Source**	**Clone**	**Catalog #**
Anti-mouse CD31 antibody	Cell Signaling Technology	D8V9E	77699S
Anti-phospho-STAT1 (Tyr701) antibody	Cell Signaling Technology	58D6	9167S
Anti-CAIX antibody	Abcam	EPR4151	Ab108351

Table S2: Mouse strains, cell lines, reagents, software and instruments used in the study.

**Mice stains**	**Source**	**Catalog #**
C57BL/6J	Jackson Laboratory	000664
B6.SJL-$\mathit{Ptprc}^{a}\mathit{Pepc}^{b}$/BoyJ (Ly5.1)	Jackson Laboratory	002014
B6.PL-$\mathit{Thy1}^{a}$/CyJ (Thy1.1)	Jackson Laboratory	000406
Ifngγr1$^{-/-}$, B6.129S7-$\mathit{Ifngr1}^{tm1Agt}$/J (IFNγR1KO)	Jackson Laboratory	003288
B6.Cg-Tg(Tek-cre)12Flv/J (Tie2^{Cre})	Jackson Laboratory	004128
C57BL/6N-$\mathit{Ifngr1}^{tm1.1Rds}$/J (IFNγR1$^{flox/flox}$)	Jackson Laboratory	25394
B6.129(Cg)-$\mathit{Gt(ROSA)26Sor}^{tm4(ACTBtdTomato\text{-}EGFP)Luo}$/J (mTmG)	Jackson Laboratory	007676
B6.129S4-$\mathit{Ifng}^{tm3.1Lky}$/J (GREAT)	Jackson Laboratory	17581
C57BL/6-Tg(TcraTcrb)1100Mjb/J	Jackson Laboratory	003831
**Reagents and column**	**Source**	**Catalog #**
eBioscience™ Fixable Viability Dye eFluor™ 455UV	Thermo Fisher Scientific	65-0868-18
RPMI1640 medium	Thermo Fisher Scientific	11875-093
Fetal bovine serum	Gemini	100-106
Penicillin-Streptomycin (10,000 U/mL penicillin, 10,000 µg/mL streptomycin)	Thermo Fisher Scientific	15140-122
Phosphate-buffered saline, without calcium and magnesium	Corning	21-040-CV
2-mercaptoethanol	Sigma	M3148-100 ml
Ovalbumin (OVA) peptide (SIINFEKL)	AnaSpec	AS-60193-1
Human interleukin-2	Peprotech	200-02
Lymphocyte Separation Medium	Promo Cell GmbH	C-44010
Liberase TM	Millipore Sigma	5401119001
Ethylenediaminetetraacetic acid	Phoenix BioTechnologies	2004
Foxp3/Transcription Factor Staining Buffer Kit	Thermo Fisher Scientific	00-5523-00
M-PER Mammalian Protein Extraction Reagent	Thermo Fisher Scientific	78501
Protease inhibitor cocktail	Crystalgen	225-015
Opal Automation IHC Kit	Akoya Bioscience	NEL871001KT
Sucrose	Fluka Analytical	B4097
Paraformaldehyde	Thermo Fisher Scientific	J19943.K2

Mounting medium containing DAPI	Thermo Fisher Scientific	00-4959-52
Serum bovine albumin	Millipore Sigma	A9418-50G
IRDye 800CW NHS ester	LI-COR Biosciences	929-70020
PD-10 Desalting Column	Cytiva	17085101
**Software and instruments**	**Source**	**Version**
FlowJo software	BD Life Sciences	v10.8
Image J software	National Institutes of Health	Fiji
ZEN software	Carl Zeiss	3.2
GraphPad Prism software	GraphPad Software	10.0.2 (232)
CytoFLEX LX flow cytometer	Beckman Coulter	N/A
ZEISS LSM 880 laser scanning confocal microscope	Carl Zeiss	N/A
Bond RXm autostainer	Leica Biosystems	N/A
Pearl^{TM} Imager	LI-COR Biosciences	N/A
Mantra Quantitative Pathology Workstation with the Inform Image Analysis Software	Perkin Elmer	Inform 2.5

Supplementary figures

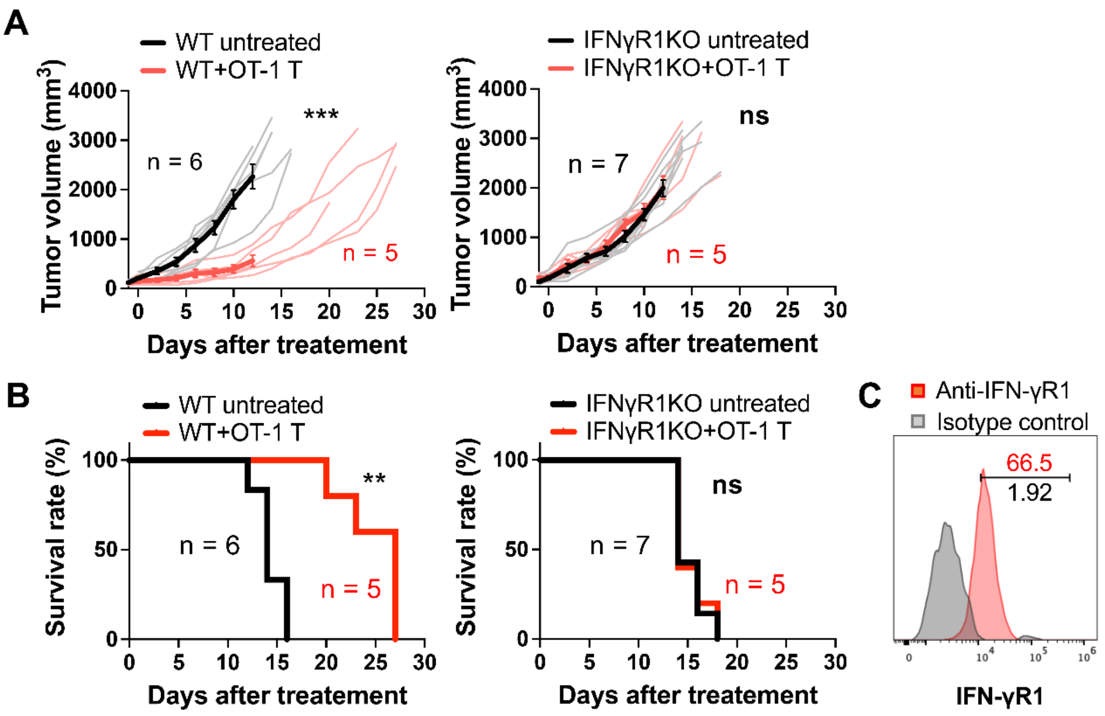

Figure S1. Requirement for IFN-γR1 expression on non-tumor cells for the tumor suppression in ACT.

A, ACT suppressed the growth of MOC2-SIINFEKL oral squamous cell carcinoma tumors in WT mice but failed to show efficacy in IFNγR1KO mice. Thin line: volume of individual tumor, bold line: average tumor volume. ***: $P < 0.001$; ns, not significant, by repeated-measure two-way ANOVA.

B, ACT prolonged survival of WT mice bearing MOC2-SIINFEKL tumors but failed in IFNγR1KO mice. **: $P < 0.01$; ns, not significant, by log-rank (Mantel–Cox) test.

C, Flow cytometry analysis indicated expression of IFN-γR1 in MOC2-SIINFEKL tumors *in vivo* (representative data of n = 3).

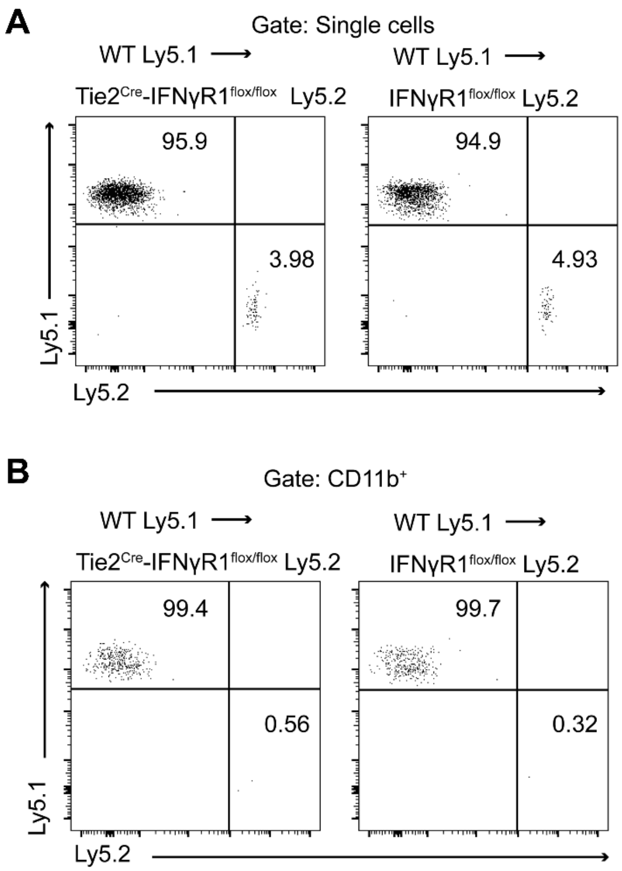

Figure S2. Successful reconstitution of peripheral blood mononuclear cells with donor-derived cells in the bone marrow chimera mice.

A, Flow cytometric analysis of Ly5.1 and Ly5.2 expression in peripheral blood mononuclear cells in Tie2^{cre}IFNγR1$^{flox/flox}$ and IFNγR1$^{flox/flox}$ mice (expressing Ly5.2) transferred with the bone marrow from Ly5.1 WT mice (WT Ly5.1 → Tie2^{cre}IFNγR1$^{flox/flox}$ Ly5.2 and WT Ly5.1 → IFNγR1$^{flox/flox}$ Ly5.2, respectively). Analyses were performed 6 weeks after bone marrow transfer (n = 3, representative data). Gated on single live cells.

B, Data in **A** were further analyzed by gating on CD11b$^+$ myeloid cell populations.

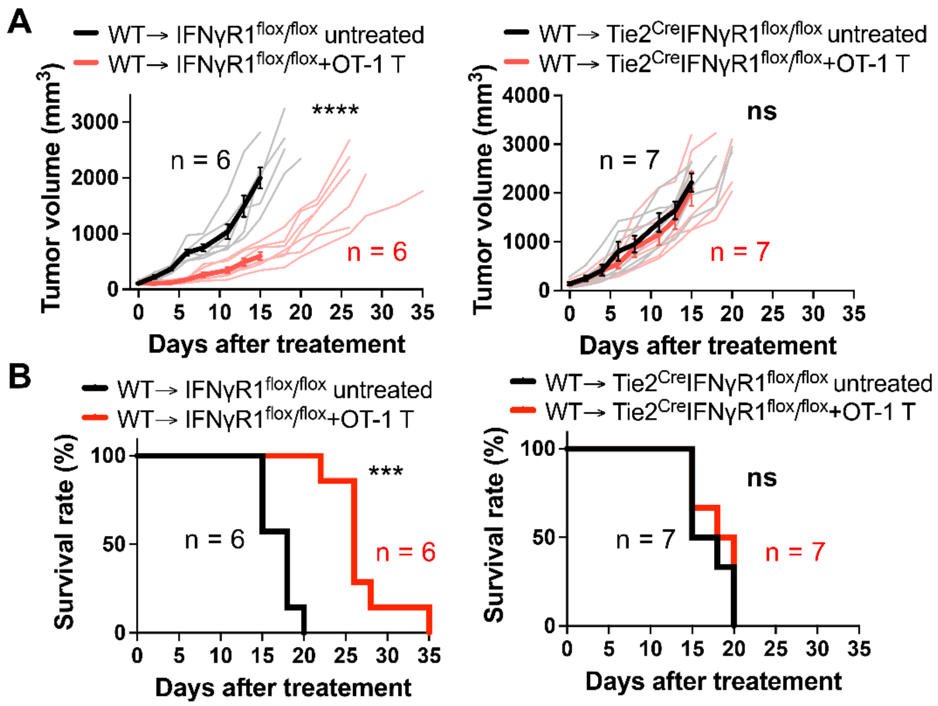

Figure S3. Requirement for IFN-γR1 expression on endothelial cells for the tumor suppression in ACT.

A, ACT suppressed MOC2-SIIFEKL tumor growth in control WT→IFNγR1$^{flox/flox}$ bone marrow chimera mice but failed to demonstrate efficacy in WT → Tie2^{cre}IFNγR1$^{flox/flox}$ bone marrow chimera mice that lack IFN-γR1 in endothelial cells. Thin line: volume of individual tumor, bold line: average tumor volume. ****: P < 0.0001; ns: not significant, by repeated-measure two-way ANOVA.

B, Prolonged survival following ACT was observed in control WT → IFNγR1$^{flox/flox}$ bone marrow chimeras bearing MOC2-SIINFEKL tumors but was abrogated in WT → Tie2^{cre}IFNγR1$^{flox/flox}$ bone marrow chimeras. ***: P < 0.001; ns, not significant, by log-rank (Mantel–Cox) test.

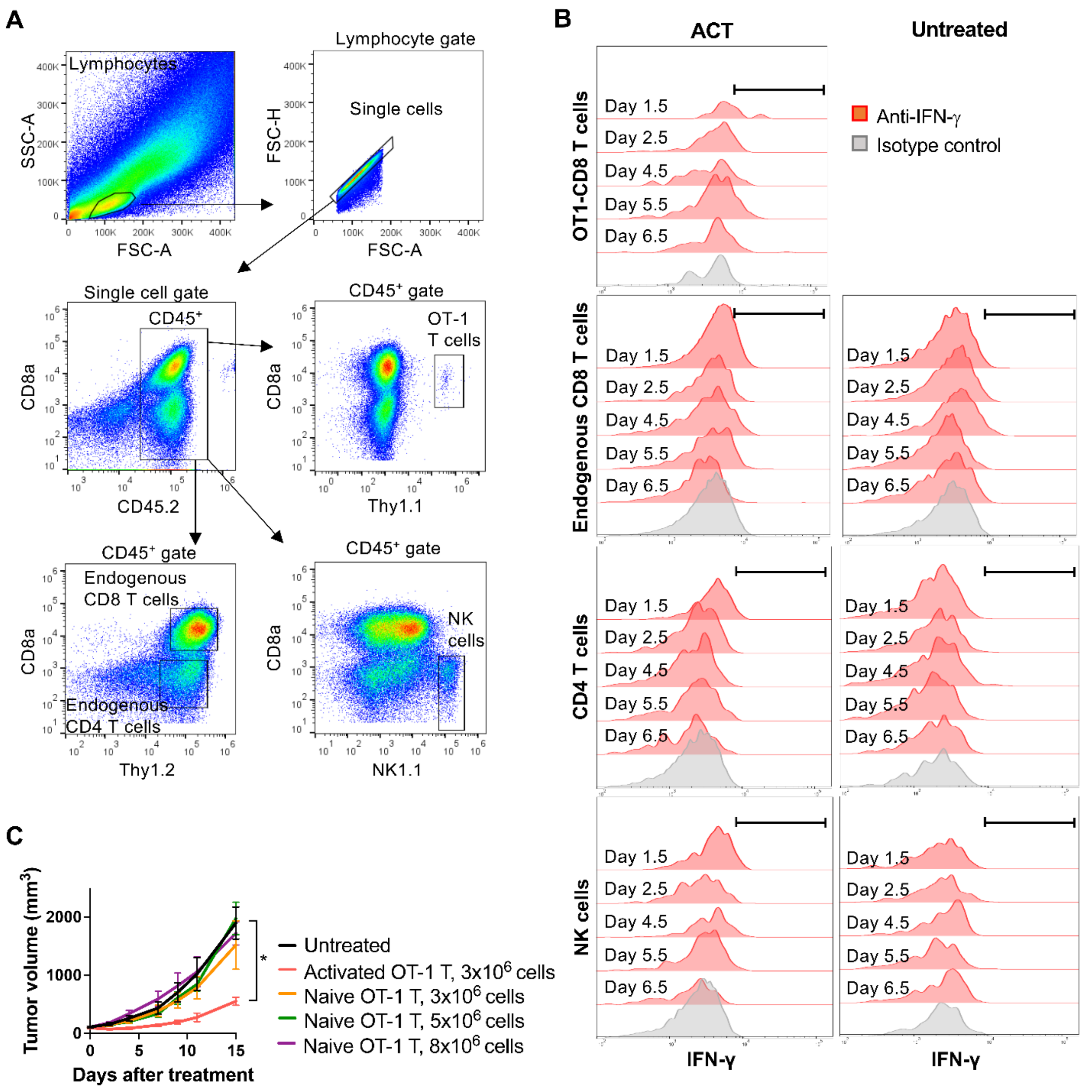

Figure S4. Levels of IFN-γ production in transferred activated OT-1 CD8 T cells and endogenous lymphocytes in the tumor after ACT, and ineffective therapy by naïve OT-1 CD8 T cells.

A, Representative gating strategy.

B, Representative flow cytometry data of IFN-γ expression levels in transferred *ex vivo* activated OT-1 CD8 T cells and endogenous CD8 T, CD4 T and NK cells collected from MCA-205-OVA-GFP tumors

indicated days after ACT or without ACT ($n = 3$, except for Day 6.5 untreated group that had $n = 2$). Marker represents IFN-γ^+ fraction.

C, Naïve OT-1 CD8 T cells failed to suppress MCA-205-OVA-GFP tumor growth even with increasing doses of cells, in contrast to activated OT-1 CD8 T cells in WT mice. $n = 3$ for the activated OT-1 T cell groups, $n = 4$ for other groups. *: $p < 0.05$, analyzed by repeated-measure two-way ANOVA comparing to the untreated group.

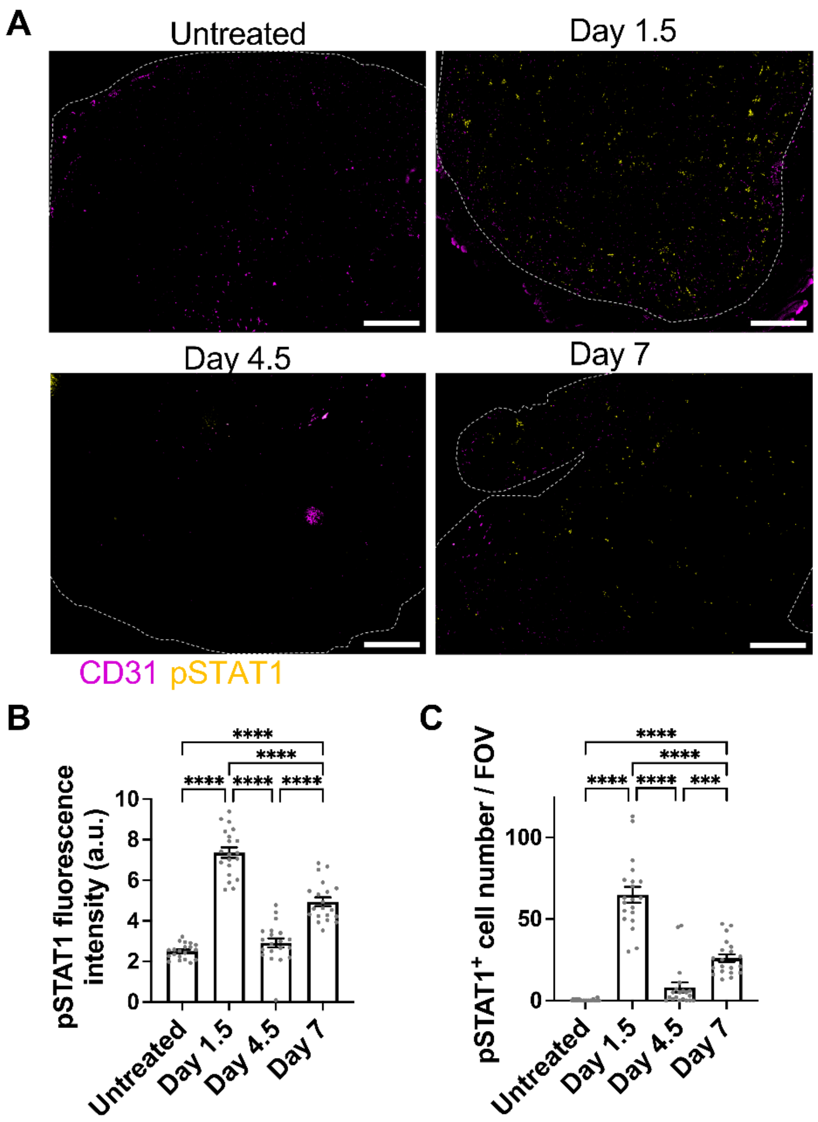

Figure S5. Induction of IFN-γ/STAT1 signaling in the tumor.

A, Representative immunofluorescence images of MCA-205-OVA-GFP tumor tissue sections in the untreated group and Day 1.5, Day 4.5, and Day 7 after ACT (n = 2), Scale bar: 500 µm.

B, Quantitation of pSTAT1-fluorescence intensity of the immunofluorescence images obtained in **A**. Each symbol indicates the average pSTAT1 fluorescence intensity in a FOV. Two tumors, 10 FOVs per tumor, were analyzed per group. ****: $P < 0.0001$, by one-way ANOVA.

C, Similarly, pSTAT1$^+$ cell number in each FOV is summarized. Each symbol indicates individual FOV and 10 FOVs per tumor, two tumors per group, were analyzed. ***: P < 0.001; ****: P < 0.0001, by one-way ANOVA.

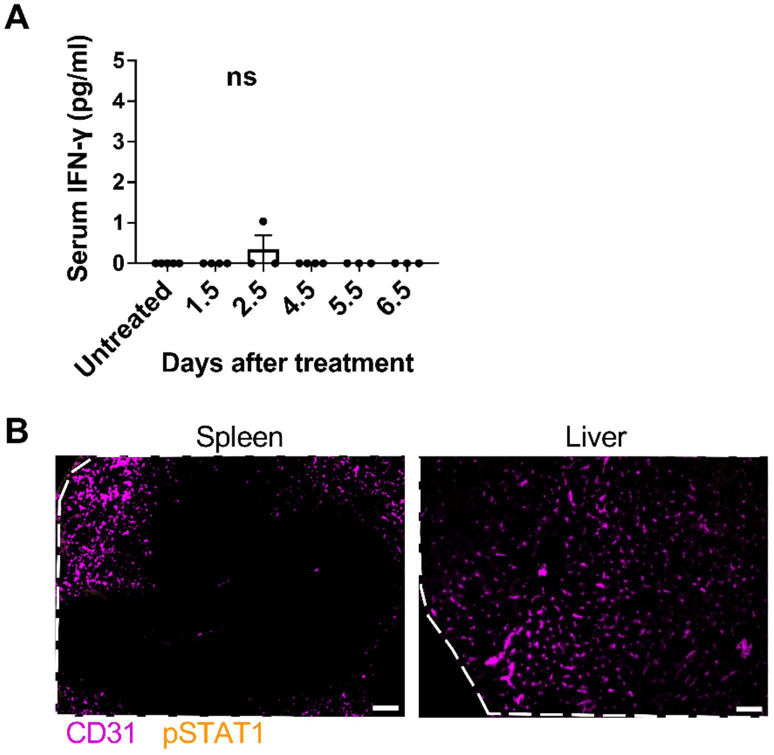

Figure S6. Minimal effect of IFN-γ on normal endothelial cells.

A, ELISA revealed minimal or undetectable IFN-γ levels in the serum at different time points after T cell transfer. n = 3 -5. ns, not significant in the comparison of any two groups by one-way ANOVA.

B, Representative immunofluorescence images of liver and spleen tissue sections on Day 1.5 in the ACT group (n = 3). No pSTAT1 was detected. White dashed lines indicate tissue boundaries. Scale bar: 50 µm.

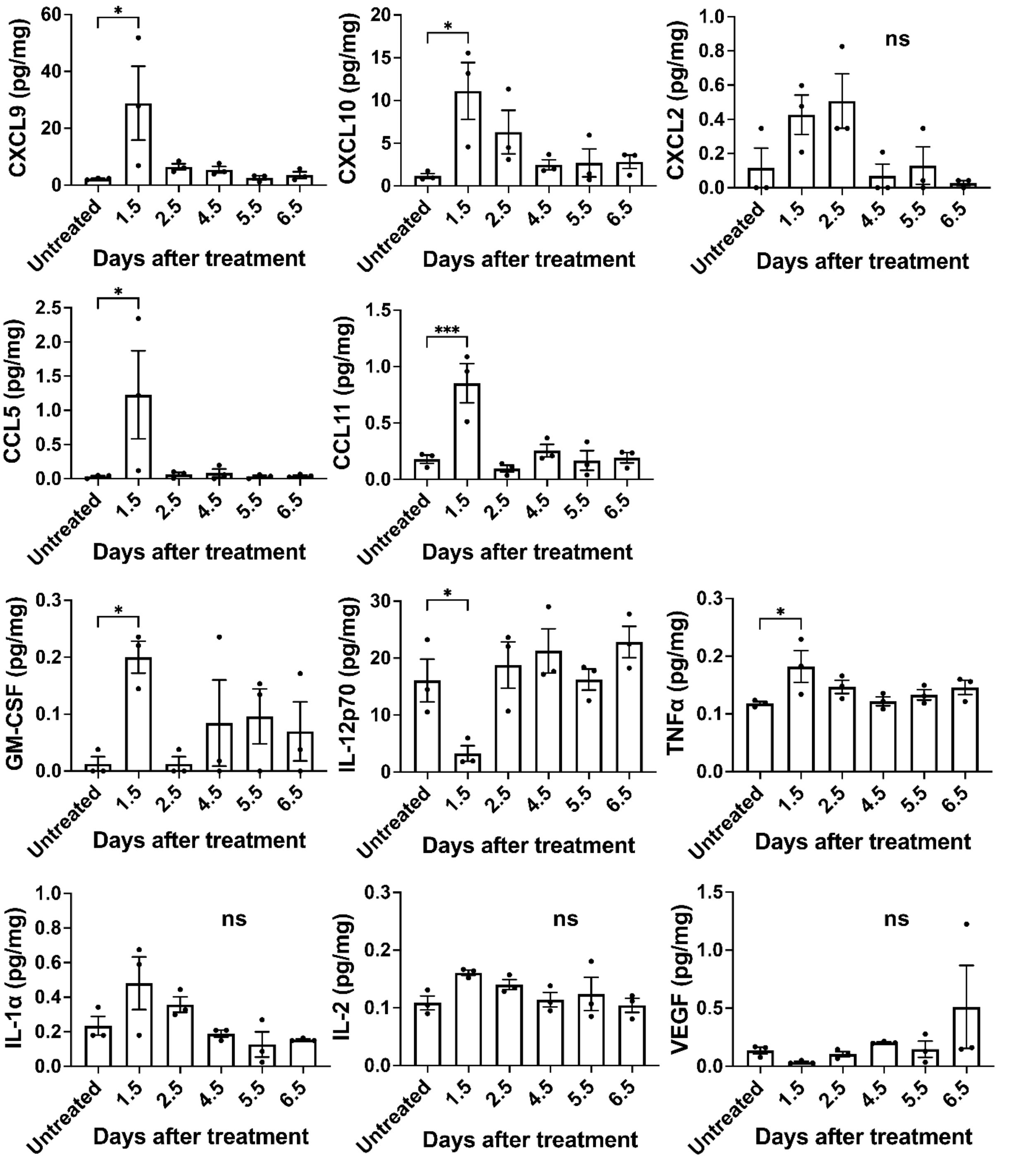

Figure S7. Changes in cytokine/chemokine expressions in the tumor microenvironment after ACT. Changes in indicated cytokine and chemokine concentrations in MCA-205-OVA-GFP tumors were analyzed at indicated time points after ACT (n = 3). One-way ANOVA was used to compare the values in the treatment groups against the untreated control. *: $p < 0.05$, ***: $p < 0.001$, ns: not significant.

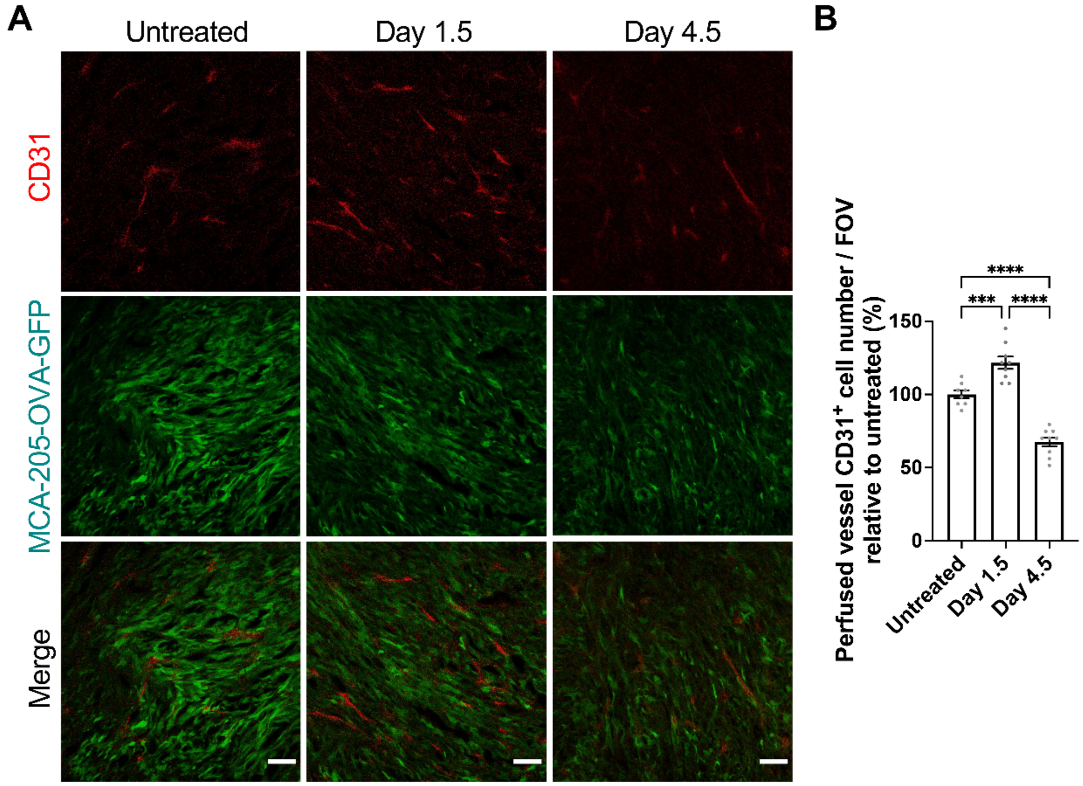

Figure S8. Decreased functional tumor blood vessels after ACT.

A, APC-conjugated anti-CD31 antibody was intravenously infused to mice bearing MCA-205-OVA-GFP tumor untreated or Days 1.5 and 4.5 after ACT. Endothelial cells in the vessels with perfusion were stained with the anti-CD31 antibody. Representative immunofluorescence images of tumor tissue sections without *ex vivo* staining (n = 3). Scale bar: 50 µm.

B, Quantitation of images obtained in **A**. Each symbol represents $CD31^+$ cell number associated with vessels with perfusion observed per FOV. Three FOVs from 2 tumors and two FOVs from one tumor were analyzed in the untreated group, and three FOVs per tumor, 3 tumors were analyzed for Day 1.5 and Day 4.5 after ACT. ***: $P < 0.001$; ****: $P < 0.0001$, by one-way ANOVA.

Legends for supplementary movies

Movie S1: A 3D video generated from the representative intravital microcopy images shown in Figure 4B, demonstrating OT-1 T cell interaction with endothelial cells at Day 1.5 after ACT. mTmG-OT-1 CD8 T cells expressing Tdtomato (magenta) were transferred to MCA-205-OVA-GFP tumor (green)-bearing mice. mTmG-OT-1 CD8 T cells were observed inside or near tumor vessels (red) that were labeled *in vivo* with Alexa Fluor 647-conjugated anti-CD31 antibody.

Movie S2: A 3D video demonstrating infiltration of mTmG-OT-1 CD8 T cells (magenta) into the MCA-205-OVA-GFP tumor (GFP) microenvironment, away from vessels (CD31, red) at Day 5 post-ACT. Tumor vessels were barely detectable. Refer to Figure 4B.

Movie S3: A 3D video illustrating significant infiltration of mTmG-OT-1 CD8 T cells (magenta) into the MCA-205-OVA-GFP tumor (GFP) microenvironment, away from vessels (CD31, red) at Day 7 post-ACT. Narrow vessels appeared on Day 7. Refer to Figure 4B.
